# Exploiting historical agronomic data to develop genomic prediction strategies for early clonal selection in the Louisiana sugarcane variety development program

**DOI:** 10.1002/tpg2.20545

**Published:** 2024-12-30

**Authors:** Dipendra Shahi, James Todd, Kenneth Gravois, Anna Hale, Brayden Blanchard, Collins Kimbeng, Michael Pontif, Niranjan Baisakh

**Affiliations:** ^1^ School of Plant, Environmental and Soil Sciences Louisiana State University Agricultural Center Baton Rouge Louisiana USA; ^2^ Sugarcane Research Unit USDA‐ARS Houma Louisiana USA; ^3^ Sugar Research Station Louisiana State University Agricultural Center St. Gabriel Louisiana USA

## Abstract

Genomic selection can enhance the rate of genetic gain of cane and sucrose yield in sugarcane (*Saccharum* L.), an important industrial crop worldwide. We assessed the predictive ability (PA) for six traits, such as theoretical recoverable sugar (TRS), number of stalks (NS), stalk weight (SW), cane yield (CY), sugar yield (SY), and fiber content (Fiber) using 20,451 single nucleotide polymorphisms (SNPs) with 22 statistical models based on the genomic estimated breeding values of 567 genotypes within and across five stages of the Louisiana sugarcane breeding program. TRS and SW with high heritability showed higher PA compared to other traits, while NS had the lowest. Machine learning (ML) methods, such as random forest and support vector machine (SVM), outperformed others in predicting traits with low heritability. ML methods predicted TRS and SY with the highest accuracy in cross‐stage predictions, while Bayesian models predicted NS and CY with the highest accuracy. Extended genomic best linear unbiased prediction models accounting for dominance and epistasis effects showed a slight improvement in PA for a few traits. When both NS and TRS, which can be available as early as stage 2, were considered in a multi‐trait selection model, the PA for SY in stage 5 could increase up to 0.66 compared to 0.30 with a single‐trait model. Marker density assessment suggested 9091 SNPs were sufficient for optimal PA of all traits. The study demonstrated the potential of using historical data to devise genomic prediction strategies for clonal selection early in sugarcane breeding programs.

## Core Ideas

- We used 12 years of historical phenotypic data of Louisiana sugarcane to devise genomic prediction strategies.
- Cross‐stage prediction validated the strategy of early clonal selection for enhancing the rate of genetic gain.
- Multi‐trait genomic selection with stalk number and sucrose at the early stage improved sugar yield prediction accuracy.
- We found that 9091 markers were sufficient for optimal prediction of most traits studied.

**Abbreviations**
BCI: bottom coincidence index
BGLR: Bayesian generalized linear regression
BL: Bayesian Lasso
BLUE: best linear unbiased estimates
BLUP: best linear unbiased prediction
BMTM: Bayesian multi‐trait model
BRR: Bayesian ridge regression
CY: cane yield
GBLUP: genomic best linear unbiased prediction
GEBV: genomic estimated breeding value
GP: genomic prediction
GRM: genomic relationship matrix
GS: genomic selection
GWAS: genome‐wide association study
MAS: marker‐assisted selection
ML: machine learning
MTA: marker‐trait association
MTGS: multi‐trait genomic selection
MVGBLUP: multi‐variate genomic best linear unbiased prediction
NS: number of stalks
PA: predictive ability
QTL: quantitative trait locus
RF: random forest
RKHS: reproducing kernel Hilbert space
rrBLUP: ridge regression best linear unbiased prediction
SNP: single nucleotide polymorphism
SVM: support vector machine
SW: stalk weight
SY: sugar yield
TCI: top coincidence index
TP: training population
TRS: theoretical recoverable sugar

## 1 INTRODUCTION

Sugarcane (*Saccharum* interspecific hybrids L.) is significant economically as an industrial crop grown in tropical and subtropical regions around the world (Deomano et al., 2020; Fickett et al., 2019; Mahadevaiah et al., 2021). It accounts for more than 70% of the total global sugar production (Satpathy et al., 2022). Although most sugarcane is used as a raw material for sugar and bioethanol, it is also used for animal feed, alcoholic beverages, fertilizer, and paper manufacturing (Islam et al., 2021; Yadav et al., 2020). In the United States, sugarcane was planted in 372,000 ha that produced 32.97 million metric tons of cane, contributing to ∼45% of the sucrose production (https://quickstats.nass.usda.gov/results/09DF6AFA-31DD-31D4-B72A-07CB4D653940). It is the number one row crop in Louisiana, with 1.82 million metric tons of sugar produced from 193,600 ha of sugarcane area harvested out of 203,200 ha planted, contributing $1.436 billion to the producers, processors, and landlords in 2022 (https://www.nass.usda.gov/Statistics_by_State/Louisiana/Publications/Crop_Releases/Crop_Production_Monthly/2022/lacropdec22.pdf).

Genetics has played a significant role in improving sugar yield (SY) components of sugarcane in the previous several decades (Hayes et al., 2021; Yadav et al., 2020). In Louisiana, sugar production has doubled in the last 50 years, primarily due to the development of improved, high‐yielding varieties combined with improved cultural management practices (Blanchard et al., 2024; Hale et al., 2022). However, the rate of genetic gain has not significantly improved in recent years (Wei & Jackson, 2016). Conventional breeding based on phenotype‐based recurrent selection takes up to 13 years from crossing to variety release (Voss‐Fels et al., 2021). The lengthy breeding cycle and narrow genetic base of modern elite cultivars are major limiting factors to improving genetic gain in sugarcane (Raboin et al., 2008). Many economic traits in sugarcane, such as cane yield (CY) and sucrose content, are complex and quantitatively (polygenically) inherited (Hoang et al., 2015; Yadav et al., 2021) with low narrow‐sense heritability (single‐plot basis), making phenotypic selection inefficient in early stages of sugarcane breeding programs (Wei & Jackson, 2016). Marker‐assisted selection (MAS), which was touted as a replacement strategy for time‐ and labor‐consuming phenotypic selection in various crops (Collard & Mackill, 2008), has been slower in sugarcane compared to other crops except for a few disease resistance traits (c.f. Satpathy et al., 2022) due to the lack of reliable, large‐effect quantitative trait loci (QTLs) and linked molecular markers for most agronomic traits (Gouy et al., 2013). Genomics research in sugarcane has been slow largely due to its large (∼10 Gb), aneupolyploid complex genome (2*n* = 8–14*x* = 100–144) resulting from the cross between *Saccharum officinarum* (2*n* = 80; *x* = 10) and *Saccharum spontaneum* (2*n* = 40–128; *x* = 8) (D'Hont, 2005; Piperidis & D'Hont, 2020).

Genomic selection (GS), since its first application in livestock breeding (Meuwissen et al., 2001), is being used as a modern genome‐wide marker‐assisted breeding method in various crop improvement programs. Unlike MAS, which utilizes a few statistically significant markers or major QTLs (Heffner et al., 2011), GS utilizes genome‐wide markers with small to large effects simultaneously in a statistical model to estimate the breeding value, known as genomic estimated breeding value (GEBV) (Calus & Veerkamp, 2011; Gouy et al., 2013). Marker effects estimated with the phenotype and genotype data of a reference or training population (TP) using statistical models are used to calculate GEBVs to predict the phenotypic performance of genotypes in the validation population based on their marker profile (Maulana et al., 2021). By enabling the selection of genotypes at earlier stages in the variety development process, GS expedites the breeding process by potentially shortening the breeding cycle and length of time for recycling parents back to the crossing stage of the recurrent selection programs, thereby accelerating the rate of genetic gain (Jannink et al., 2010).

GS has been implemented in several crops including rice (Labroo et al., 2021; Xu et al., 2021), corn (Atanda et al., 2021; Beyene et al., 2021), wheat (Guo et al., 2020), loblolly pine (Rios et al., 2021), potato (Endelman et al., 2018), and blueberry (de Bem Oliveira et al., 2019; Ferrão et al., 2021). GS provides unique opportunities for improving the rate of genetic gain for quantitative traits in sugarcane by efficient selection of genotypes based on their GEBV‐informed near‐precise ranks, and for reducing the length of breeding cycle (Deomano et al., 2020; Hayes et al., 2021) that traditionally has relied on time‐consuming, labor‐intensive, and expensive process of selecting a desirable genotype from among thousands of seedlings (Yadav et al., 2023). The decreasing cost of high‐throughput genotyping, emerging high‐throughput phenotyping, and evolving bioinformatics and statistical tools suggest immense potential for deployment of GS in sugarcane. Compared to other crops, there have been a few GS studies reported in sugarcane (Mahadevaiah et al., 2021). Gouy et al. (2013) were the first to implement GS in sugarcane using two panels of 167 genotypes each for 10 traits in breeding programs of reunion and found predictive ability (PA) ranging from 0.11 to 0.62 within panel and 0.13 to 0.55 between panels. The promising PAs with relatively small TP sizes implied that GS can be employed as an effective sugarcane breeding strategy. With three different panels consisting of a total of 2351 genotypes from both early and advanced stages of an Australian commercial sugarcane breeding program, PA ranging from 0.25 to 0.45 was observed for two sugar‐related traits, CY and commercially extractable sucrose (Deomano et al., 2020). Hayes et al. (2021) showed genomic PAs of 0.30–0.44 for CY, SY, and fiber content (Fiber) in 3984 sugarcane genotypes. In the United States, Islam et al. (2021) studied brown rust and orange rust reactions in 432 genotypes from the Florida breeding program where they showed that GS with nonparametric machine learning (ML) models outperformed the parametric models. The same authors also reported PAs between 0.11 and 0.37 for various SY traits such as Brix, fiber, pol, sucrose content, stalk diameter, stalk population, and stalk weight (SW) using 432 genotypes (Islam et al., 2022). A pilot‐scale study in Louisiana showed that the low PAs of 0.23 for sucrose yield and 0.19 for CY without fixed effect covariates due to soil and crop type were attributed to a relatively smaller TP size (Satpathy et al., 2022).

The existence of allelic and nonallelic (dominance and epistasis) interactions controlling the traits in sugarcane poses a challenge to the implementation of GS. Studying CY, SY, and Fiber in 3006 sugarcane genotypes, Yadav et al. (2021) showed an improvement in PA through extended genomic best linear prediction (eGBLUP) model that factored nonadditive genetic effects. High allele dosage at a given locus also complicates GS in sugarcane breeding (Voss‐Fels et al., 2021). Accounting for allelic dosage information improved the accuracy of genomic prediction (GP) in polyploid species, including potato (Endelman et al., 2018) and blueberry (de Bem Oliveira et al., 2020), although it can increase genotyping costs for higher depth of coverage to capture all the alleles and computational complexity.

**Core Ideas**

- We used 12 years of historical phenotypic data of Louisiana sugarcane to devise genomic prediction strategies.
- Cross‐stage prediction validated the strategy of early clonal selection for enhancing the rate of genetic gain.
- Multi‐trait genomic selection with stalk number and sucrose at the early stage improved sugar yield prediction accuracy.
- We found that 9091 markers were sufficient for optimal prediction of most traits studied.

Several statistical models with varying accuracy, computational complexity, and requirements have been developed and implemented in GS. Examples of parametric methods are genomic best linear unbiased prediction (GBLUP), ridge regression best linear unbiased prediction (rrBLUP), and Bayesian methods (Endelman, 2011; Habier et al., 2011; VanRaden, 2008). Both GBLUP and rrBLUP assume normal distribution and equal variance for marker effects (Endelman, 2011), whereas Bayesian models assume varying effects and variances resulting in increased shrinkage for small single nucleotide polymorphism (SNP) variation effects and less shrinkage on large SNP effects (Habier et al., 2010). In contrast, ML models have lenient assumptions for the normality and variance of markers, and they utilize nonlinear kernels to capture complex and nonlinear interactions between markers and traits (Ogutu et al., 2011; Ryo & Rillig, 2017; Zhao et al., 2020). In the present study, we evaluated 22 different GS models of which some accounted for additive, dominance, and epistatic interactions; fixed effect markers; multiple traits; and so on using commercial and elite parental genotypes having 12 years of phenotypic data from the Louisiana commercial sugarcane breeding program and compared PAs for sucrose and CY traits within and across different selection stages.

## 2 MATERIALS AND METHODS

### 2.1 Plant materials and traits

The experimental materials used in this study were sugarcane genotypes used and/or developed by the sugarcane breeding programs of the Louisiana State University (LSU) Agricultural Center and Agricultural Research Service of the United States Department of Agriculture (USDA). Data for six traits, theoretical recoverable sugar (TRS), number of stalks (NS), SW, CY, SY, and Fiber, were obtained in both plant cane (P) and ratoon crops—first (R1) and second (R2) ratoon from four different stages of sugarcane variety development, namely, second line (stage 2), nursery (stage 3), infield (stage 4), and outfield (stage 5) trials over 12 years (2010–2021). The soil type of experimental locations was categorized into heavy‐textured (clay; H) type and light‐textured (sandy loam; L) type. Stage 2 trials were conducted in 4.88‐m long plots at two locations (one H and one L). Stage 3 experiments were conducted at eight locations (four each of H and L) in plots of 4.88‐m and 6.10‐m long for on‐station and off‐station nurseries, respectively. Stage 4 trials were carried out in 7.32‐m long plots at seven locations (2 H and 5 L). Stage 5 trial was conducted in 15.24‐m long plots at 14 locations (7 H and 7 L). The experiments were performed in a complete randomized block design with two replications for stages 3 and 4, and three replications for stage 5 trials. Stage 2 genotypes were not replicated within a location but repeated in locations and years. Two commercial varieties (L 01–299 and HoCP 96–540) were used as checks for all the trials. An augmented design was followed in stage 2 where the checks were repeated thrice in each row and location/year that accounted for environmental variations. A total of 567 genotypes were used in the study with both phenotype and genotype information for stages 2 (212), 3 (218), 4 (100), and 5 (37).

The NS per plot was taken before harvesting. In each plot, 10 stalks were harvested and weighed to determine the SW in stages 2 and 3 and estimate TRS (sucrose content, kg Mg^−1^). CY (Mg ha^−1^) was calculated as the product of NS and SW. In stages 4 and 5, plots were harvested by a chopper harvester that emptied into a single‐axle, high‐dump wagon equipped with electronic load cells to record plot weight, which was used to estimate CY. The harvested stalks were run through a Spectracane near‐infrared spectroscopic system (Bruker Corporation) to estimate TRS. Fiber (fiber %) was measured as described by Bischoff and Gravois (2003). Alternatively, for the trials conducted by the USDA Sugarcane Research Unit, sucrose was estimated using the methods described by Todd et al. (2024). Stalks from stages 2 and 4 were processed using the core press method (Legendre, 1992) to estimate TRS and Fiber, and for stages 3 and 5, TRS was estimated from juice extract from a roller mill. Lastly, SY (Mg ha^−1^) was computed by multiplying CY with TRS.

### 2.2 Analysis of variance

Phenotypic datasets were stratified in 44 different ways (Table S1) for analysis. A combined analysis was carried out with data across environments (year and location) and crop types (plant cane and ratoon crops 1 and 2). Further, phenotypic analysis was performed by clustering the environments based on soil type (H or L) and by sub‐setting data based on crop type (P, R1, and R2) to optimize the GS model for target crop and soil type. Adjusted phenotypic means (i.e., best linear unbiased estimates [BLUEs]) were estimated using the following mixed model in ASReml‐R v4 as follows:

(1) $$\begin{array}{ccc} Y_{ijkl} & = & \mu +E_{i}+R_{ij}+C_{k}+EC_{ik}+G_{l}+GC_{kl}+GE_{il}\\ & & +\;\mathrm{GC}E_{ikl}+\varepsilon_{ijkl} \end{array}$$

Where *Y_ijkl_* is the phenotype, μ is the mean effect, *E_i_* is the effect of *i*th environment (location and year), *R_ij_* is the effect of *j*th replication nested in *I* environment *j*, *C_k_* is the effect of *k*th crop type, *EC_ik_* is the effect of *i*th environment and *k*th crop interaction, *G_l_* is the effect of *l*th genotypes, GC*_kl_* is the effect of *l*th genotype by *k*th crop interaction, GE*_il_* is the interaction effect of *l*th genotype by *i*th environment, GCE*_ikl_* is the interaction effect of *l*th genotype by *k*th crop by *i*th environment, and *ε_ijkl_* is the residual effect. Pearson correlation coefficients among the traits were determined using ggcorrplot package in R.

### 2.3 Heritability

Broad‐sense heritability estimate (*H* ^2^) of the traits was calculated as the proportion of total genetic variance to total phenotypic variance. The “Ad hoc Holland” broad‐sense heritability method (Holland et al., 2002) was used to address the differences in genotypes in different environments and crop types. *H* ^2^ was calculated assuming genotype and other effects as random using the following formula:

(2) $$H^{2}=\frac{\sigma_{G}^{2}}{\sigma^{2}G+\frac{{\sigma^{2}}_{\mathrm{GE}}}{{\tilde{n}}_{E}}+\frac{{\sigma^{2}}_{\mathrm{GC}}}{{\tilde{n}}_{C}}+\frac{{\sigma^{2}}_{\mathrm{GEC}}}{{\tilde{n}}_{\mathrm{EC}}}+\frac{\sigma_{e}^{2}}{{\tilde{n}}_{\mathrm{ECr}}}}$$

where *σ* ^2^_G_, *σ* ^2^_GE_, *σ* ^2^_GC_, and *σ* ^2^_GEC_ represent genetic, genotype by environment, genotype by crop type, genotype by environment by crop type, and residual variances, respectively. Here, ${\tilde{n}}_{E}$, ${\tilde{n}}_{C}$, ${\tilde{n}}_{\mathrm{EC}}$, and ${\tilde{n}}_{\mathrm{ECr}}$ are harmonic means of genotypes associated with environment, crop, environment × crop, and residual variances, respectively. Narrow‐sense heritability was also calculated with the pedigree (*h* ^2^p) as well as marker genotype (*h* ^2^m) information (described below) using the restricted maximum likelihood approach implemented in ASReml‐R.

### 2.4 Genotyping

Genomic DNA from sugarcane leaf tissues isolated with the CTAB (cetyltrimethylammoniumbromide) miniprep method was assessed for quality and quantity using 1% agarose‐TAE (Tris‐acetate‐EDTA) gel and ND‐1000 (Nanodrop) as described earlier (Fickett et al., 2019). For Capture‐Seq based genotyping, 20,000 probes (120 bp) were designed from the sugarcane R570 mosaic monoploid genome (Garsmeur et al., 2018) based on sorghum gene models and tiled at 3x. For library preparations, genomic DNA was sheared to ∼500 bp, fragments were end‐repaired and A‐tailed, followed by incorporation of uniquely indexed Illumina adaptors and PCR (polymerase chain reaction) enrichment. Samples were pooled equimolar and sequenced on Illumina NovaSeq (2 × 150 bp) at RAPiD Genomics LLC. Raw sequence reads were demultiplexed using Illumina's BCL‐Convert, quality‐evaluated using fastp, trimmed for adaptors and length with trimmomatic, and mapped against the R570 monoploid genome with BWA. Variant (SNP) calls were performed using Freebayes (Garrison & Marth, 2012), GBS Tassel (Glaubitz et al., 2014), and Samtools (Li et al., 2009). SNPs called by all three software were further filtered based on missing data (≤20%) and minor allele frequency (≥10%). SNPs that were in complete linkage disequilibrium (LD) (*r* ^2 ^= 1) were also filtered to retain one representative SNP, resulting in 20,451 SNPs used in the study.

### 2.5 Genomic prediction

For GP, the GBLUP additive model was further extended by including dominance (D), epistatic interactions [additive‐additive (GG), additive‐dominant (GD], and genome‐wide average heterozygosity (H), which resulted in 10 different GS models. The full model is as follows:

(3) $$\begin{array}{ccc} & & \left(G+D+GD+GG+H\right):Y=\mu +\mathrm{Xb}+Z_{1}g+Z_{2}d\\ & & \;\;+\;Z_{3}\mathrm{gg}+Z_{4}\mathrm{gd}+\varepsilon \end{array}$$

where *Y* is the phenotypic response, μ is the effect of the overall mean, **b** is the vector of fixed effects, **X** is the incidence matrix of fixed effects, and **Z**_1_, **Z**_2_, **Z**_3_, and **Z**_4_ are the incidence matrices for random effects; **g** is the vector of additive effects ∼ *N* (0, **G**_A_ *σ* ^2^_A_), where **G**_A_ is the genomic relationship matrix (GRM) due to additive effect and *σ* ^2^_A_ is the additive genetic variance; **d** is the vector of dominance effects ∼ *N* (0, *G*_D_ *σ* ^2^_D_), where G_D_ is the GRM due to dominance effects and *σ* ^2^_D_ is dominance genetic variance, **gg** is a vector of additive‐by‐additive effects, where **gg**∼ *N* (0, *G*_AA_ *σ* ^2^_AA_) where G_AA_ is the GRM due to additive‐by‐additive effects and *σ* ^2^_AA_ is the additive‐by‐additive variance; **gd** is the vector of additive‐by‐dominance effects **gd**∼ *N* (0, *G*_AD_ *σ* ^2^_AD_), where G_AD_ is the GRM due to additive‐by‐dominance effects and *σ* ^2^_AD_ is the additive‐by‐dominance variance; and the residual effect *ε* ∼ *N*(0, **I** *σ* ^2^_e_) where **I** is the identity matrix and *σ* ^2^_e_ is the residual variance components. Additive (A) (VanRaden, 2008), dominance (D) (Vitezica et al., 2013), and pedigree and genomic information combined (hybrid matrix; H) (Legarra et al., 2009) matrices were constructed using R‐package AGHmatrix (Amadeu et al., 2016). Covariance matrices due to epistasis, additive‐additive (GG), and additive‐dominance (GD) were computed using Hadamard products of **G** and **D** matrices. The estimated average genome‐wide heterozygosity (Miller et al., 2014) was incorporated in the above model as fixed effects. AsReml‐R was used to perform eGBLUP models. An rrBLUP model was run in the rrBLUP package (Endelman, 2011). Six Bayesian methods (BL, Bayesian ridge regression [BRR], reproducing kernel Hilbert space [RKHS], Bayes A, Bayes B, and Bayes C) were implemented with Bayesian generalized linear regression (BGLR) package (Pérez & de los Campos, 2014) with 12,000 iterations, 2000 burn‐ins, thinning of five, and default hyper‐parameters. In addition, two nonparametric ML methods, random forest (RF) and support vector machine (SVM), were utilized. The RF method employed the randomForest package in R (Cutler et al., 2022) and the SVM method was implemented with a radial kernel and epsilon regression using the R package e1071 (Meyer et al., 2019). Altogether, 22 different prediction models were employed for GP.

### 2.6 Genome‐wide association study

Genome‐wide association study (GWAS) was performed using an enriched compressed mixed linear model executed in Genome Association Prediction Integrated Tool in R (Lipka et al., 2012) using 19 datasets (nine from S2 and five each from S3 and S4) for each trait to identify significant marker‐trait associations (MTAs). Significant SNPs (*p* < 0.001) from each dataset were used as fixed effect covariates in the prediction model GBLUP for a particular trait/dataset.

### 2.7 Cross‐validation and PA

First, a fivefold cross‐validation approach was undertaken within 15 selected datasets for the three stages (2, 3, and 4). Genotypes within each stage were randomly divided into five subsets with four subsets used as the TP to predict the fifth subset. Then a cross‐stage approach was undertaken where genotypes from stage 2 served as the training set to predict the performance of the genotypes at stage 5 as the validation population. The PA was assessed by the Pearson correlation coefficient between GEBVs and observed phenotypes (BLUEs). All GS models were compared using both cross‐validation methods. Further, to evaluate the effectiveness of the GS models in identifying top‐ and bottom‐performing genotypes in the validation set, the coincidence index for the 20% top (top coincidence index [TCI]) and bottom (bottom coincidence index [BCI]) performing genotypes was computed as described earlier (Fernandes et al., 2018; Hamblin & Zimmermann, 1986).

### 2.8 Effect of marker density

To determine the effect of marker density on GS, nine different LD‐based schemes of marker filters were evaluated using markers with *r* ^2 ^< 0.1 (2792 markers), *r* ^2 ^< 0.15 (4399 markers), *r* ^2 ^< 0.2 (6112 markers), *r* ^2 ^< 0.3 (9091markers), *r* ^2 ^< 0.4 (11,613 markers), *r* ^2 ^< 0.6 (15,592 markers), *r* ^2 ^< 0.8 (18,012 markers), and *r* ^2 ^< 0.99 (20,451 markers). Fivefold cross‐validation was carried out with the dataset from stage 2 in plant cane under light soil (S2PL).

### 2.9 Multi‐trait GS

Considering that SY is a compound trait dependent on five other traits, multi‐trait genomic selection (MTGS) for SY was performed using multi‐variate genomic best linear unbiased prediction (MVGBLUP) in ASReml‐R and Bayesian multi‐trait model (BMTM) in R package BGLR. The model was run with various combinations of other correlated traits as responses to predict SY. Only the phenotype for SY was hidden in the validation population. The MTGS schemes used genotypic information from both training and validation populations and phenotypic data of correlated traits in validation population. The S2PL data were used for within‐stage fivefold cross‐validation and as a training set for cross‐stage validation to predict the performance of genotypes at stage 5 in plant cane under light soil (S5PL) using the model:

(4) y=μ+Zα+ε

where **y** is the vector of BLUEs for t traits; μ is the overall mean effect; **Z** is the incidence matrix; *α* is the genotypic predictor ∼MVN (0, **Σ** ⊗ K), where **Σ** is the variance–covariance matrix across traits, *K* is the realized additive GRM among individuals estimated from the markers; and **ε** is the residual errors vector ∼MVN (0, **R** ⊗ I), where **R** is the variance–covariance matrix for the residual effects for each individual among traits and  **I** is the identity matrix. ⊗ is the Kronecker product of two matrices. **Σ** was estimated as an unstructured matrix and **R** as a diagonal matrix.

## 3 RESULTS

### 3.1 Phenotypic summary statistics, trait correlation, and heritability

The average TRS over 12 years ranged from 93.10 Kg Mg^−1^ in stage 2, first ratoon crop under heavy soil (S2R1H) to 119.89 Kg Mg^−1^ in stage 5, first ratoon under light soil (S5R1L) (Table S1). SY averaged between 6.78 Mg ha^−1^ at stage 5 in second ratoon under heavy soil (S5R2H) and 16.65 Mg ha^−1^ at S2PL. Likewise, for CY traits, NS had a minimum value of 64,048 in stage 4, plant cane under heavy soil (S4PH), and a maximum of 134,057 in stage 2, second ratoon under light soil (S2R2L). SW ranged between 0.73 kg in stage 5, second ratoon, heavy soil (S5R2H), and 1.22 kg in S2PL. The mean CY values spanned from 59.08 Mg ha^−1^ (S5R2H) to 142.00 Mg ha^−1^ (S2PL). Fiber values ranged from 8.30 (S5PL) to 13.59 (S5R2L).

Stage‐wise data revealed that all the traits showed significant variation within a stage depending on the crop and soil type (Table S1). Cross‐stage comparisons showed that the highest mean values of NS, SW, CY, and SY were observed, as expected in small unreplicated trials in stage 2, which decreased with the advancement of the stage. The TRS, as expected with selection during advancement, showed an increasing trend with the evaluation stage. On the other hand, the Fiber was somewhat inconsistent until S4 and comparatively lower in S5. Such inconsistency in stages 2 and 5 could be due to different methods of estimation between the LSU and USDA breeding programs as described in the methods section.

#### 3.1.1 Correlation between traits

**FIGURE 1:**
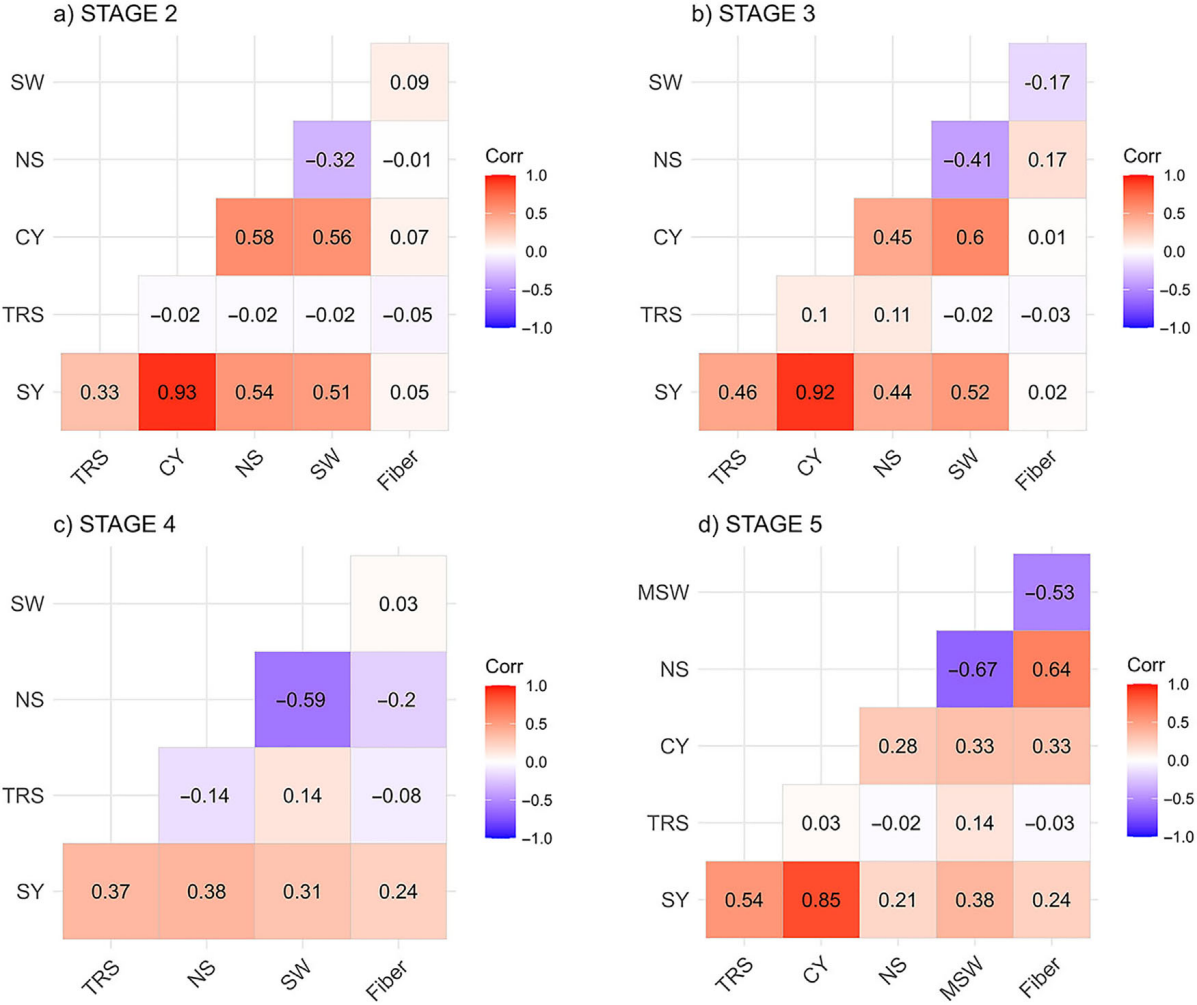
Correlation among six cane and sugar yield traits in stage 2 (S2), stage 3 (S3), stage 4 (S4), and stage 5 (S5). CY, cane yield (Mg ha^−1^); Fiber, fiber content (%); NS, number of stalks (ha^−1^); SW, stem weight (Mg); SY, sugar yield (Mg ha^−1^); TRS, theoretical recoverable sugar (kg Mg^−1^).

Overall, SY had a significant positive correlation with TRS (0.33 at stage 2 to 0.54 at stage 5), CY (0.85 at stage 5 to 0.93 at stage 2), NS (0.21 at stage 5 to 0.54 at stage 2), and SW (0.31 at stage 4 to 0.52 stage 3) (Figure 1). Correlation of SY with Fiber was low (0.02 to 0.24) but significant (Figure 1, Table 1). Correlations of TRS with CY, NS, SW, and Fiber were non‐significant with both positive and negative values, except at stage 3 where it showed significant positive correlation with NS (0.11). Expectedly, CY had significant positive correlation with NS (0.28 to 0.58), SW (0.25 to 0.60), and Fiber (0.01 to 0.33). NS had a significant negative correlation with SW (−0.67 at stage 5 to −0.32 at stage 2), whereas it had both negative and positive correlation with Fiber (−0.20 to 0.64). The correlation between SW and Fiber was also inconsistent (−0.53 to 0.09). Similar trend was observed in datasets across stage, crop, and soil type, where SY exhibited a significant positive correlation with TRS (0.20–0.56), NS (0.04–0.71), and the highest with CY (0.76–0.97) (Table S2). SY mostly displayed a positive correlation with SW. Correlation between CY and TRS was either significant or nonsignificant with negative or positive values. Likewise, CY displayed a significant positive correlation with NS, and SW for the most part. SW and NS were significantly negatively correlated at −0.17 to −0.92.

**TABLE 1:** Correlation among six cane and sugar yield traits at different selection stages across crop and soil type.

Stage 2						
	SY	TRS	CY	NS	MSW	Fiber
SY	1.00					
TRS	0.33^**^	1.00				
CY	0.93^**^	−0.02	1.00			
NS	0.54^**^	−0.02	0.58^**^	1.00		
SW	0.51^**^	−0.02	0.56^**^	−0.32^**^	1.00	
Fiber	0.05	−0.05	0.07	−0.01	0.09	1.00

Stage 3						
	SY	TRS	CY	NS	MSW	Fiber
SY	1.00					
TRS	0.46^**^	1.00				
CY	0.92^**^	0.10	1.00			
NS	0.44^**^	0.11	0.45^**^	1.00		
SW	0.52^**^	−0.02	0.60^**^	−0.41^**^	1.00	
Fiber	0.02	−0.03	0.01	0.18	−0.17	1.00

Stage 4						
	SY	TRS	CY	NS	MSW	Fiber
SY	1.00					
TRS	0.37^**^	1.00				
CY	0.89^**^	−0.09	1.00			
NS	0.39^**^	−0.14	0.49^**^	1.00		
SW	0.31^**^	0.14	0.25^*^	−0.59^**^	1.00	
Fiber	0.24^*^	−0.08	0.29^*^	−0.20	0.03	1.00

Stage 5						
	SY	TRS	CY	NS	MSW	Fiber
SY	1.00					
TRS	0.54^**^	1.00				
CY	0.85^**^	0.03	1.00			
NS	0.21	−0.02	0.28^*^	1.00		
SW	0.38^**^	0.14	0.33^**^	−0.67	1.00	
Fiber	0.24^*^	−0.03	0.33	0.64	−0.53	1.00

Abbreviations: CY, cane yield (Mg ha^−1^); MSW, mean stem weight (Mg); NS, number of stalks (ha^−1^); SY, sugar yield (Mg ha^−1^); TRS, theoretical recoverable sugar; Fiber, fiber content (%).

*p < 0.05. **p < 0.01.

#### 3.1.2 Heritability of the traits

The broad sense heritability (*H* ^2^) and narrow sense heritability based on pedigree (*h* ^2^p) and markers (*h* ^2^m) of six traits showed a wide range of variation in different stages and crop/soil combinations (Table S3). In general, *H* ^2^ for all the traits was low at stage 2 and then increased at advanced stages with no significant differences among the traits. The variation in *H* ^2^ for the traits did not follow a definitive pattern for crop and/or soil type, which makes it difficult to identify locations within a stage that consistently provides reproducible data. The *H* ^2^ of SY and CY were comparable to each other at all stages with combined data (Table S3). Interestingly for the combined dataset, NS showed a moderately high *H* ^2^ ranging from 0.53 at stage 2 to 0.76 at stage 5. SW exhibited higher values of *H* ^2^ with 0.64 at stage 2 to 0.84 at stage 3 for the combined dataset, closely followed by TRS. Surprisingly, Fiber had low *H* ^2^ at stages 3 (0.15) and 4 (0.27) stage compared to moderately high values at stages 2 (0.68) and 5 (0.78). The *h* ^2^m values were higher for all traits compared to *h* ^2^p at all stages except at stage 2 where it was the opposite (*h* ^2^p > *h* ^2^m). SW and TRS consistently displayed higher *h* ^2^m values ranging from 0.45 to 0.77 and 0.45 to 0.59 at S3 and S5, respectively, except for the trial at S2 (0.18 for SW and 0.23 for TRS) as compared to other traits. For Fiber, the *h* ^2^m was the highest (0.58) only at S5. Traits such as CY and SY demonstrated lower *h* ^2^m compared to other traits.

### 3.2 Prediction models performance

#### 3.2.1 Within stage cross‐validation

**FIGURE 2:**
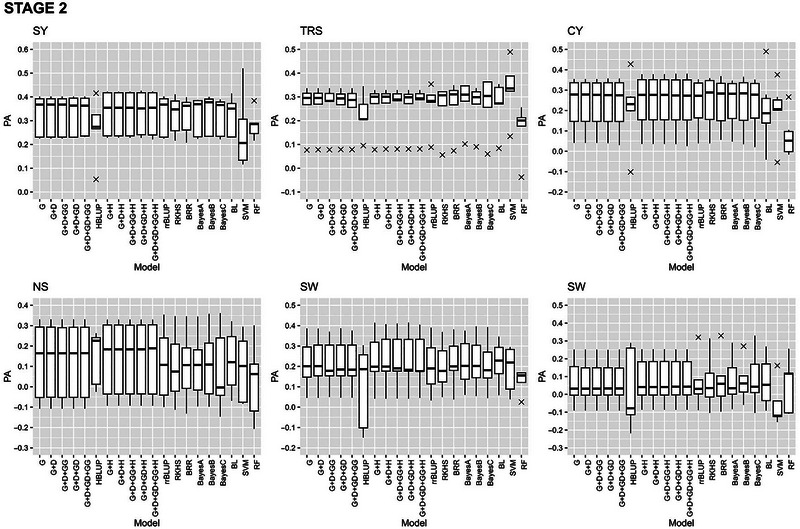
Predictive ability (PA) of six cane and sugar yield traits in stage 2 (S2). BL, Bayesian Lasso; BRR, Bayesian ridge regression; CY, cane yield (Mg ha^−1^); D, dominance; Fiber, fiber content (%); G, GBLUP; GG, additive‐additive; GD, additive‐dominance; H, heterozygosity; HBLUP, hybrid matrix; NS, number of stalks (ha^−1^); RF, random forest; RKHS, reproducing kernel Hilbert space; rrBLUP; ridge regression BLUP; SVM, support vector machine; SW, stem weight (Mg); SY, sugar yield (Mg ha^−1^); TRS, theoretical recoverable sugar (kg Mg^−1^).

**FIGURE 3:**
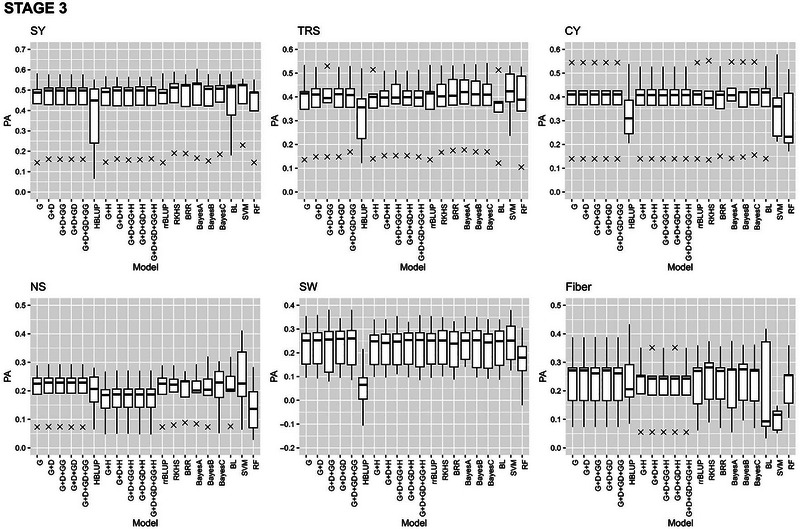
Predictive ability (PA) of six cane and sugar yield traits in stage 3 (S3) dataset. BL, Bayesian Lasso; BRR, Bayesian ridge regression; CY, cane yield (Mg ha^−1^); D, dominance; Fiber, fiber content (%); G, GBLUP; GG, additive‐additive; GD, additive‐dominance; H, heterozygosity; HBLUP, hybrid matrix; NS, number of stalks (ha^−1^); RF, random forest; RKHS, reproducing kernel Hilbert space; rrBLUP; ridge regression BLUP; SVM, support vector machine; SW, stem weight (Mg); SY, sugar yield (Mg ha^−1^); TRS, theoretical recoverable sugar (kg Mg^−1^).

**FIGURE 4:**
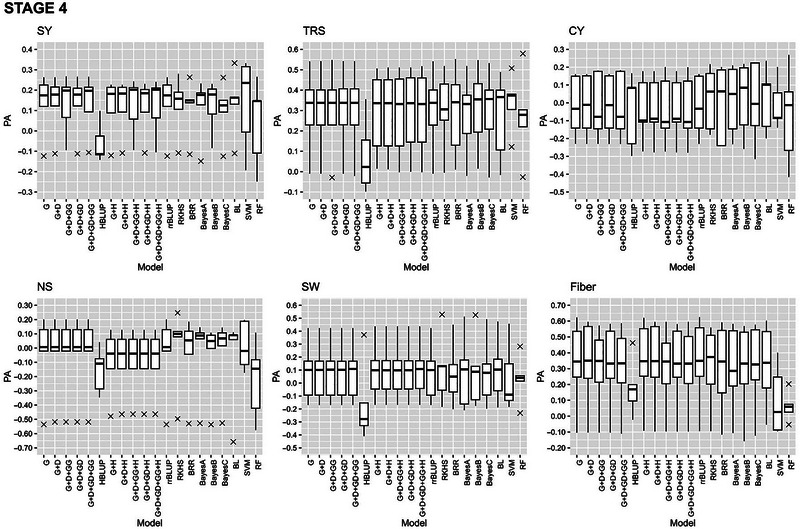
Predictive ability (PA) of six cane and sugar yield traits in stage 4 (S4). BL, Bayesian Lasso; BRR, Bayesian ridge regression; CY, cane yield (Mg ha^−1^); D, dominance; Fiber, fiber content (%); G, GBLUP; GG, additive‐additive; GD, additive‐dominance; H, heterozygosity; HBLUP, hybrid matrix; NS, number of stalks (ha^−1^); RF, random forest; RKHS, reproducing kernel Hilbert space; rrBLUP; ridge regression BLUP; SVM, support vector machine; SW, stem weight (Mg); SY, sugar yield (Mg ha^−1^); TRS, theoretical recoverable sugar (kg Mg^−1^).

The GP models in stage 2, stage 3, and stage 4 exhibited varying PAs for the six traits with fivefold cross validations of the reference populations (Figures 2, 3, 4).

##### Stage 2

The PA for SY in combined datasets at stage 2 (S2) were not significantly different (0.32–0.33) for the parametric models except for HBLUP (0.27) (Figure 2, Table 2). The ML method SVM recorded the lowest PA (0.26). However, the maximum coincidence index (CI) of 0.34 was achieved with SVM for the top 20% performing clones (TCI), while RF recorded the highest CI for the bottom 20% performing clones (BCI). For TRS, RF predicted with the highest accuracy at 0.33. All the best linear unbiased prediction (BLUP methods showed similar PA (0.26) except for HBLUP (0.22). While BRR had the highest TCI (0.31), G + D had the highest (0.35) BCI. The rrBLUP model had the highest PA (0.25) for CY. For CY, the highest value of TCI and BCI was predicted by (G + D) (0.26) and RF (0.31), respectively. Several BLUP methods showed PA values of 0.14 for NS and 0.23 for SW. Bayesian Lasso (BL) method showed the highest value of PA (0.09) for Fiber.

**TABLE 2:** Predictive ability (PA) of six cane and sugar yield traits in stage 2 across crop and soil type.

	SY			TRS			CY			NS			SW			Fiber		
Model	PA	TCI	BCI	PA	TCI	BCI	PA	TCI	BCI	PA	TCI	BCI	PA	TCI	BCI	PA	TCI	BCI
G	0.32	0.26	0.30	0.26	0.28	0.26	0.24	0.12	0.28	0.13	0.20	0.21	0.22	0.30	0.20	0.07	0.26	0.17
G + D	0.32	0.26	0.30	0.26	0.28	0.35	0.24	0.26	0.28	0.13	0.18	0.32	0.22	0.34	0.22	0.07	0.11	0.15
G + D + GG	0.32	0.26	0.30	0.26	0.28	0.25	0.23	0.12	0.28	0.13	0.20	0.21	0.21	0.29	0.20	0.07	0.26	0.17
G + D + GD	0.32	0.26	0.30	0.26	0.28	0.25	0.23	0.12	0.28	0.13	0.20	0.21	0.22	0.31	0.18	0.07	0.27	0.17
G + D + GD + GG	0.33	0.26	0.30	0.25	0.28	0.25	0.23	0.12	0.26	0.13	0.20	0.21	0.21	0.31	0.20	0.07	0.27	0.17
G + H	0.33	0.28	0.28	0.26	0.26	0.25	0.24	0.14	0.26	0.14	0.20	0.25	0.23	0.30	0.21	0.08	0.27	0.17
G + D + H	0.33	0.28	0.28	0.26	0.24	0.25	0.24	0.14	0.26	0.14	0.20	0.25	0.23	0.30	0.21	0.08	0.27	0.17
G + D + GG + H	0.33	0.28	0.28	0.26	0.26	0.25	0.24	0.14	0.26	0.14	0.20	0.25	0.23	0.30	0.21	0.08	0.27	0.17
G + D + GD + H	0.33	0.28	0.28	0.26	0.26	0.25	0.24	0.14	0.26	0.14	0.20	0.25	0.23	0.30	0.21	0.08	0.27	0.17
G + D + GD + GG + H	0.33	0.28	0.28	0.26	0.26	0.25	0.24	0.14	0.24	0.14	0.20	0.25	0.23	0.30	0.21	0.08	0.27	0.17
HBLUP	0.27	0.29	0.32	0.22	0.27	0.26	0.21	0.24	0.25	0.14	0.18	0.27	0.10	0.20	0.16	0.03	0.19	0.21
rrBLUP	0.32	0.26	0.30	0.25	0.26	0.26	0.25	0.14	0.28	0.13	0.20	0.21	0.23	0.30	0.28	0.09	0.27	0.17
RKHS	0.32	0.27	0.30	0.26	0.24	0.25	0.24	0.14	0.27	0.11	0.16	0.26	0.20	0.33	0.22	0.07	0.25	0.18
BRR	0.32	0.26	0.30	0.25	0.31	0.23	0.24	0.12	0.26	0.10	0.14	0.26	0.20	0.30	0.20	0.07	0.23	0.15
BayesA	0.32	0.26	0.30	0.27	0.24	0.23	0.24	0.12	0.26	0.10	0.14	0.26	0.22	0.30	0.20	0.08	0.25	0.19
BayesB	0.33	0.26	0.30	0.28	0.26	0.23	0.24	0.12	0.29	0.10	0.14	0.28	0.22	0.33	0.24	0.08	0.25	0.17
BayesC	0.32	0.26	0.32	0.26	0.28	0.27	0.24	0.13	0.29	0.11	0.14	0.28	0.22	0.33	0.18	0.08	0.27	0.15
BL	0.32	0.27	0.28	0.27	0.27	0.25	0.23	0.14	0.26	0.08	0.14	0.25	0.21	0.30	0.18	0.09	0.25	0.20
Non‐parametric																		
RF	0.28	0.22	0.35	0.33	0.28	0.26	0.20	0.12	0.31	0.09	0.21	0.21	0.18	0.29	0.18	−0.05	0.24	0.14
SVM	0.26	0.34	0.30	0.26	0.26	0.29	0.20	0.19	0.28	0.13	0.24	0.23	0.22	0.28	0.22	0.07	0.25	0.21

Abbreviations: BCI, bottom coincidence index; BRR, Bayesian ridge regression; BL, Bayesian Lasso; CY, cane yield (Mg ha^−1^); D, dominance; Fiber, fiber content (%); G, GBLUP; GG, additive‐additive; GD, additive‐dominance; H, heterozygosity; HBLUP, hybrid matrix; NS, number of stalks (ha^−1^); RF, random forest; RKHS, reproducing kernel Hilbert space; rrBLUP; ridge regression BLUP; SW, stem weight (kg); SY, sugar yield (Mg ha^−1^); TCI, top coincidence index; TRS, theoretical recoverable sugar (kg Mg^−1^); SVM, support vector machine.

When S2 data were stratified by crop and soil type, the highest PA was recorded for SW (0.31) by both BRR and SVM in plant cane crops under light soil (S2PL) (Table S4). RF showed the highest CI for top (0.45) and RKHS for bottom (0.42) performing clones for CY and NS, respectively. Under heavy soil conditions (S2PH), Bayesian models predicted with the highest accuracy both SY (BL) and SW (BayesA) at 0.23, while the best TCI was recorded for CY (0.48) by SVM (Table S5). BayesA also had the maximum PA for SY (0.39) in ratoon crop under light soil (S2RL), whereas RKHS and RF had the highest TCI for TRS (0.44) and BCI for TRS (0.39), respectively (Table S6). For ratoon crop under heavy soil conditions, S2RH (Table S7), BRR had the highest PA for SY (0.39), but both TCI (0.42) and BCI (0.59) were the best for Fiber by the eGBLUP methods.

##### Stage 3

In the stage 3 combined (S3) dataset, the highest PA was 0.24 for NS and 0.45 for SY (Figure 3, Table 3). All models except HBLUP showed comparable PA for SY (0.41–0.45), CY (0.36–0.38), and SW (0.17–0.25). For TRS, both RF and SVM performed better with the highest (0.44) PA followed by Bayes A (0.40). Similar to SY (0.45), SVM also showed the highest prediction accuracies for NS (0.24) and SW (0.25). The highest PA value for CY was 0.38 with Bayes C. Both ML models scored the highest TCI and BCI, both at 0.44 for TRS, while SVM outperformed others with the highest TCI (0.39) for SY. Model G + D predicted the top‐performing clones with the highest TCI (0.35) for NS, whereas Bayes A had the highest TCI of 0.38 for SW. Bayesian models performed better than other models with higher PA for Fiber.

**TABLE 3:** Predictive ability (PA) of six cane and sugar yield traits in stage 3 across crop and soil type.

	SY			TRS			CY			NS			SW			Fiber		
	PA	TCI	BCI	PA	TCI	BCI	PA	TCI	BCI	PA	TCI	BCI	PA	TCI	BCI	PA	TCI	BCI
G	0.43	0.37	0.31	0.37	0.40	0.35	0.38	0.29	0.33	0.20	0.29	0.25	0.23	0.32	0.20	0.24	0.26	0.29
G + D	0.43	0.37	0.31	0.37	0.39	0.37	0.38	0.32	0.24	0.21	0.35	0.26	0.23	0.35	0.26	0.24	0.20	0.12
G + D + GD	0.44	0.37	0.29	0.38	0.35	0.35	0.38	0.29	0.33	0.21	0.29	0.27	0.23	0.33	0.24	0.24	0.26	0.29
G + D + GG	0.44	0.36	0.29	0.38	0.34	0.33	0.38	0.29	0.33	0.21	0.27	0.27	0.22	0.33	0.22	0.23	0.26	0.29
G + D + GD + GG	0.44	0.37	0.29	0.38	0.34	0.35	0.38	0.29	0.33	0.21	0.29	0.27	0.23	0.36	0.22	0.23	0.26	0.29
G + H	0.43	0.38	0.31	0.36	0.38	0.33	0.37	0.29	0.31	0.17	0.27	0.24	0.22	0.34	0.20	0.22	0.26	0.31
G + D + H	0.43	0.34	0.31	0.37	0.38	0.33	0.37	0.29	0.31	0.17	0.27	0.24	0.22	0.36	0.24	0.22	0.26	0.31
G + D + GD + H	0.43	0.34	0.33	0.37	0.38	0.33	0.37	0.29	0.31	0.17	0.27	0.24	0.22	0.33	0.24	0.22	0.26	0.31
G + D + GG + H	0.43	0.34	0.31	0.38	0.38	0.35	0.37	0.29	0.31	0.17	0.27	0.24	0.23	0.34	0.26	0.22	0.26	0.31
G + D + GD + GG + H	0.43	0.34	0.31	0.37	0.34	0.33	0.37	0.29	0.31	0.17	0.27	0.24	0.22	0.36	0.24	0.22	0.26	0.31
Hmat	0.36	0.31	0.32	0.32	0.28	0.27	0.34	0.32	0.35	0.19	0.24	0.21	0.06	0.22	0.20	0.24	0.19	0.31
RR_20K	0.43	0.37	0.31	0.37	0.40	0.35	0.38	0.29	0.33	0.20	0.29	0.25	0.23	0.32	0.20	0.23	0.26	0.30
RKHS	0.45	0.34	0.35	0.38	0.38	0.33	0.37	0.29	0.29	0.21	0.25	0.25	0.23	0.32	0.19	0.24	0.24	0.30
BayesA	0.45	0.36	0.31	0.40	0.36	0.37	0.38	0.29	0.30	0.20	0.26	0.25	0.24	0.38	0.22	0.22	0.24	0.29
BayesB	0.44	0.36	0.29	0.39	0.36	0.33	0.38	0.29	0.29	0.20	0.27	0.27	0.23	0.32	0.20	0.24	0.26	0.31
BayesC	0.45	0.37	0.35	0.39	0.34	0.35	0.38	0.29	0.29	0.21	0.29	0.27	0.22	0.30	0.20	0.23	0.24	0.30
BL	0.44	0.36	0.38	0.35	0.42	0.35	0.38	0.25	0.33	0.21	0.25	0.27	0.22	0.32	0.22	0.20	0.26	0.29
BRR	0.45	0.34	0.33	0.39	0.38	0.40	0.37	0.29	0.28	0.20	0.27	0.27	0.22	0.30	0.19	0.23	0.24	0.29
Non‐parametric																		
RF	0.41	0.32	0.26	0.44	0.44	0.44	0.31	0.31	0.31	0.14	0.21	0.22	0.17	0.28	0.19	0.23	0.24	0.31
SVM	0.45	0.39	0.37	0.44	0.44	0.44	0.36	0.33	0.30	0.24	0.25	0.25	0.25	0.36	0.20	0.10	0.18	0.24

Abbreviations: BCI, bottom coincidence index; BRR, Bayesian ridge regression; BL, Bayesian Lasso; CY, cane yield (Mg ha^−1^); D, dominance; Fiber, fiber content (%); G, GBLUP; GG, additive‐additive; GD, additive‐dominance; H, heterozygosity; HBLUP, hybrid matrix; NS, number of stalks (ha^−1^); RF, random forest; RKHS, reproducing kernel Hilbert space; rrBLUP; ridge regression BLUP; SW, stem weight (kg); SY, sugar yield (Mg ha^−1^); TCI, top coincidence index; TRS, theoretical recoverable sugar (kg Mg^−1^); SVM, support vector machine.

In stage 3 plant cane crop under light soil (S3PL), the PA for SY was the highest (0.42) with one of the eGBLUP models that accounted for heterozygosity (G + D + GG + H) compared to the same model without H (0.41) (Table S8), although the highest TCI (0.41) and BCI (0.44) for CY were documented by BayesB or BL. In plant cane crop under heavy soil (S3PH), maximum PA (0.40) was observed for SY by HBLUP and RF, whereas HBLUP model had the highest TCI (0.46) for SY and BCI (0.48) for TRS (Table S9). HBLUP also had the highest PA (0.34) for SY with the dataset at stage 3 in ratoon crop under light soil (S3RL), but the highest PA was observed for TRS (0.46) by RKHS (Table S10). On the other hand, G + D was the best for both TCI and BCI for SW (0.51) and TRS (0.36), respectively. The highest PA (0.32) and BCI (0.39) were shown by the ML method SVM for Fiber in stage 3 ratoon crop under heavy soil (S3RH) and the highest TCI by an eGBLUP model for SY (0.28) (Table S11).

##### Stage 4

At stage 4 across crop and soil type, SVM showed the highest PA of 0.14 and 0.34 for SY and TRS, respectively (Figure 4, Table 4). While G + H and BL recorded the best TCI (0.44) and BCI (0.34), respectively, for TRS, RF had the highest BCI (0.26) for SY and G + D and Bayes A with the highest and similar TCI and BCI (0.42) for CY. The PA of CY (0.12) and SW (0.11) was low with the highest value recorded by RKHS. The highest PA (0.34) for Fiber was obtained by rrBLUP.

**TABLE 4:** Predictive ability (PA) of six cane and sugar yield traits in stage 4 genotypes across crop and soil type.

	SY			TRS			CY			NS			SW			Fiber		
	PA	TCI	BCI	PA	TCI	BCI	PA	TCI	BCI	PA	TCI	BCI	PA	TCI	BCI	PA	TCI	BCI
G	0.13	0.23	0.22	0.30	0.40	0.25	0.08	0.40	0.32	−0.04	0.16	0.11	0.09	0.18	0.18	0.33	0.40	0.34
G + D	0.13	0.27	0.22	0.30	0.18	0.27	0.09	0.42	0.33	−0.04	0.23	0.12	0.09	0.22	0.16	0.33	0.19	0.18
G + D + GD	0.13	0.27	0.22	0.30	0.40	0.25	0.09	0.40	0.37	−0.04	0.16	0.11	0.09	0.18	0.18	0.32	0.32	0.34
G + D + GG	0.13	0.26	0.22	0.30	0.40	0.25	0.08	0.36	0.32	−0.04	0.16	0.11	0.09	0.18	0.18	0.30	0.37	0.34
G + D + GD + GG	0.13	0.23	0.22	0.30	0.40	0.25	0.08	0.36	0.29	−0.04	0.16	0.11	0.09	0.18	0.18	0.30	0.37	0.34
G + H	0.12	0.23	0.22	0.29	0.44	0.26	0.06	0.40	0.29	−0.09	0.20	0.11	0.10	0.18	0.22	0.34	0.37	0.34
G + D + H	0.12	0.23	0.22	0.29	0.44	0.26	0.06	0.40	0.29	−0.09	0.20	0.11	0.10	0.18	0.22	0.33	0.37	0.34
G + D + GD + H	0.12	0.23	0.22	0.29	0.44	0.26	0.06	0.40	0.29	−0.09	0.20	0.11	0.10	0.18	0.22	0.32	0.32	0.34
G + D + GG + H	0.12	0.23	0.22	0.28	0.44	0.26	0.06	0.37	0.29	−0.09	0.20	0.11	0.10	0.18	0.22	0.30	0.40	0.34
G + D + GD + GG + H	0.12	0.19	0.22	0.29	0.44	0.26	0.06	0.37	0.29	−0.09	0.20	0.11	0.10	0.18	0.22	0.31	0.33	0.34
Hmat	−0.06	0.15	0.11	0.16	0.27	0.23	0.06	0.20	0.29	−0.15	0.15	0.20	−0.16	0.11	0.18	0.18	0.34	0.22
rrBLUP	0.13	0.23	0.22	0.30	0.40	0.25	0.08	0.40	0.32	−0.04	0.16	0.11	0.09	0.18	0.18	0.34	0.37	0.34
RKHS	0.12	0.33	0.22	0.30	0.40	0.30	0.12	0.37	0.41	0.01	0.16	0.11	0.11	0.23	0.18	0.31	0.35	0.34
BayesA	0.11	0.27	0.22	0.28	0.40	0.26	0.11	0.40	0.42	−0.02	0.16	0.15	0.08	0.18	0.18	0.30	0.36	0.34
BayesB	0.12	0.23	0.22	0.31	0.40	0.22	0.12	0.40	0.38	−0.06	0.16	0.11	0.09	0.22	0.14	0.30	0.32	0.38
BayesC	0.10	0.23	0.22	0.29	0.40	0.30	0.10	0.37	0.38	−0.04	0.16	0.08	0.09	0.23	0.14	0.31	0.31	0.30
BL	0.14	0.23	0.22	0.27	0.31	0.34	0.12	0.40	0.41	−0.06	0.20	0.19	0.10	0.18	0.18	0.32	0.23	0.30
BRR	0.12	0.24	0.22	0.29	0.40	0.26	0.09	0.37	0.37	−0.04	0.16	0.11	0.07	0.18	0.14	0.30	0.37	0.34
Non‐parametric																		
RF	0.04	0.19	0.26	0.27	0.39	0.22	0.03	0.31	0.33	−0.22	0.20	0.03	0.03	0.18	0.08	0.06	0.43	0.20
SVM	0.14	0.27	0.21	0.34	0.39	0.22	0.09	0.25	0.41	0.02	0.28	0.08	0.04	0.23	0.14	0.10	0.30	0.14

Abbreviations: BCI, bottom coincidence index; BRR, Bayesian ridge regression; BL, Bayesian Lasso; CY, cane yield (Mg ha^−1^); D, dominance; Fiber, fiber content (%); G, GBLUP; GG, additive‐additive; GD, additive‐dominance; H, heterozygosity; HBLUP, hybrid matrix; NS, number of stalks (ha^−1^); RF, random forest; RKHS, reproducing kernel Hilbert space; rrBLUP; ridge regression BLUP; SW, stem weight (kg); SY, sugar yield (Mg ha^−1^); TCI, top coincidence index; TRS, theoretical recoverable sugar (kg Mg^−1^); SVM, support vector machine.

The highest PA was demonstrated by Bayesian model, BRR, for TRS (0.31), whereas BayesA had the best TCI and BCI for TRS (0.44) and Fiber (0.43), respectively, in the stage 4 plant cane under light soil (S4PL) (Table S12). Under heavy soil plant cane (S4PH), the highest PA (0.62) was achieved with Bayesian models (BayesA, BayesB, and BRR) as well as RF for SW. However, the PA for SY was the lowest 0.07 (Table S13). The BayesB model had the highest CI for the top‐performing clones for TRS (0.33), whereas SVM had the maximum CI for bottom 20% clones for Fiber (0.37). In ratoon crop under light soil (S4RL), RF exhibited the highest PA at 0.46 for SY but both TCI and BCI were recorded for Fiber by BL (0.47) and HBLUP (0.39), respectively (Table S14). In the ratoon crop under heavy soil (S4RH), the highest PA was obtained with RF for Fiber (0.45), closely followed by Bayes C for TRS (0.44) and HBLUP for SW (0.43) (Table S15), while the maximum TCI and BCI both at 0.58 were recorded by RKHS for SW and RF for Fiber, respectively.

#### 3.2.2 Cross‐stage GP

Cross‐stage GPs were made for the performance of the clones in advanced stages of the breeding trials as the testing population based on the genomic breeding values estimated from the clonal performance at the early stage 2 in plant cane crop. When S2 (stage 2 combined across crop and soil type) data were used to predict stage 5 combined (S5), plant cane combined (S5P), and ratoon combined (S5R) (Table S16), ML methods predicted with the highest accuracy of 0.44 (RF) for TRS in S5, 0.33 (RF) for SW in S5R, and 0.51 (SVM) for SY and 0.30 (SVM) for CY in S5P. For SY in S5, Bayes B had the highest PA (0.32) and TCI (0.44), otherwise, RF recorded maximum PA for SW (0.29) and Fiber (0.01) by RF and SVM (same as BayesC for CY [0.24] in S5). In both S5P and S5R, ML models again had the highest PA for SY, TRS, CY, SW, and Fiber, except TRS (0.37) by HBLUP in S5R.

With S2P as the predictor, BRR and SVM recorded the highest PA (0.49) for SY, SVM for TRS (0.42), and BRR for CY (0.34) in clones at S5, S5P, and S5R, respectively (Table S17). RKHS showed the highest PA for SW (0.46) and TCI for SY (0.57) in S5, whereas SVM predicted top‐performing clones for TRS (0.52) in S5P.

When S2L was used to predict clones for S5PL, S5R1L, and S5R2L (Table S18), the highest PA at 0.31 was shown by HBLUP in S5PL and by Bayesian models (RKHS and BL) in S5R1L for TRS. CY was best predicted with SVM (0.29) in S5PL. RF had the highest ability to predict SW (0.41) in S5R2L and Fiber (0.46) in S5R1L. With S2PL data as the training set to predict clones for S5PL, S5R1L, and S5R2L (Table S19), HBLUP method showed the maximum PA for SY (0.59), CY (0.61), NS (0.54), and Fiber (0.50), while RF performed the best with the highest PA of 0.24 for TRS and SVM for SW (0.32) in S5PL. PAs of the traits for clones were generally less in ratoon crops with the highest for SW by RF at 0.22 and 0.28 in S5R1L and S5R2L. Fiber was best predicted at 0.46 accuracy by both RKHS and Bayes A closely followed by BRR (0.45) in S5R1L.

When S2H dataset was modeled to predict clones in S5PH, S5R1H, and S5R2H, SVM had the highest PA (0.39) for TRS. BRR and Fiber recorded the highest TCI (0.35) and BCI (0.38) for SY and Fiber in S5PH. The highest prediction for S5R1H was made by HBLUP for TRS (0.66), whereas BayesB and BL predicted the top‐ and bottom‐performing clones with TCI and BCI both at 0.55 for SY and CY, respectively (Table S20). In S5R2H, HBLUP showed the highest PA of 0.42 (TRS) followed by BL (0.40) for NS. BL had the highest TCI and BCI both at 0.35 for NS. The same TCI value was recorded for SY by RF, while SVM had the same highest BCI value for CY and SW. In the case where the S2PH was used to predict S5PH, S5R1H, and S5R2H (Table S21), RF proved to be the best for SW with PA at 0.35 as well as the highest TCI for Fiber (0.38) in S5PH. In both S5R1H and S5R2H, HBLUP performed the best with a PA of 0.44 and 0.43 (same as RF), respectively, for TRS. But G + D had the highest TCI of 0.55 for both TRS (same as BL for SY) and CY and SVM predicted with the highest accuracy for the bottom 25% of the clones for CY (0.55) in S5R1H. In S5R2H, all the Bayesian models except RKHS had similar TCI (0.35) and BCI (0.55) for TRS.

With S2RL dataset as the predictor for clones at S5R1L and S5R2L (Table S22), the predictions for most traits were low except BayesB and BayesC, which had the highest PA for SW (0.35) in S5R1L, while HBLUP was the best for TRS at both S5R1L (0.18) and S5R2L (0.23). While both ML models had the highest TCI (0.35) for SY and SW in S51RL, SVM had the highest BCI (0.55) for SY in S52RL. When the S2RH data were used as a predictor (Table S23), SVM showed the best PA for TRS in both S5R1H (0.39) and S5R2H (0.56), whereas RF had the highest TCI (0.55) and SVM with the highest BCI (0.35) for the same trait.

Using S2P data as the training set to predict S5R1 and S5R2, RF had the best PA (0.51) and SVM with the best TCI (0.35) for TRS at S5R1 (Table S24). The highest BCI (0.35) was recorded by both RF and SVM for Fiber. For S5R2, however, SVM had the highest PA (0.39) for TRS, whereas the top‐performing clones were best predicted by HBLUP for TRS and SVM for SW and Fiber all at 0.35. There was no difference between the models for the BCI for most traits.

Finally, when the S2R dataset was used to predict S5R1 and S5R2 (Table S25), SVM was the best with the PAs of 0.56 and 0.49 for TRS in both S5R1 and S5R2, respectively. While all models yielded negative predictions for Fiber (except RF) and NS, G + D + GD + H had the highest TCI (0.35) for Fiber and BL for NS (0.17).

Interestingly, the PA of some of the traits in some datasets increased with HBLUP that jointly considered pedigree and marker‐based information. For example, the PA increased with HBLUP for CY in S2PH and S2RH; SY in S3PH and S3RL; SW in S4RH; TRS in S5, S5R from S2, SY, CY, NS, and Fiber in S5PL from S2PL; TRS in S5PH, S5R1H, and S5R2H; and CY in S5R1H from S2H as well in some stage 4 trials.

#### 3.2.3 Significant SNP‐MTAs as fixed effects

Genome‐wide association mapping identified significant MTAs for the six traits at different stages of the breeding trials with different combinations of crop type and soil type (Table S26). MTAs common among the traits were observed at all stages except with the second stage plant cane heavy soil (S2PH) dataset. A small number of significant marker associations were identified for SY (2), TRS (4), CY (2), and Fiber (3) whereas eight and 17 markers showed associations with NS and SW, respectively, in S2PH. Similarly, except for 10 markers with TRS, four (SY and CY) or five (NS, SW, and Fiber) markers showed significant associations in S4PH. Markers unique for a trait at a given stage and condition were used as fixed effects in GBLUP model (G + S) to determine their effect on the GP.

**FIGURE 5:**
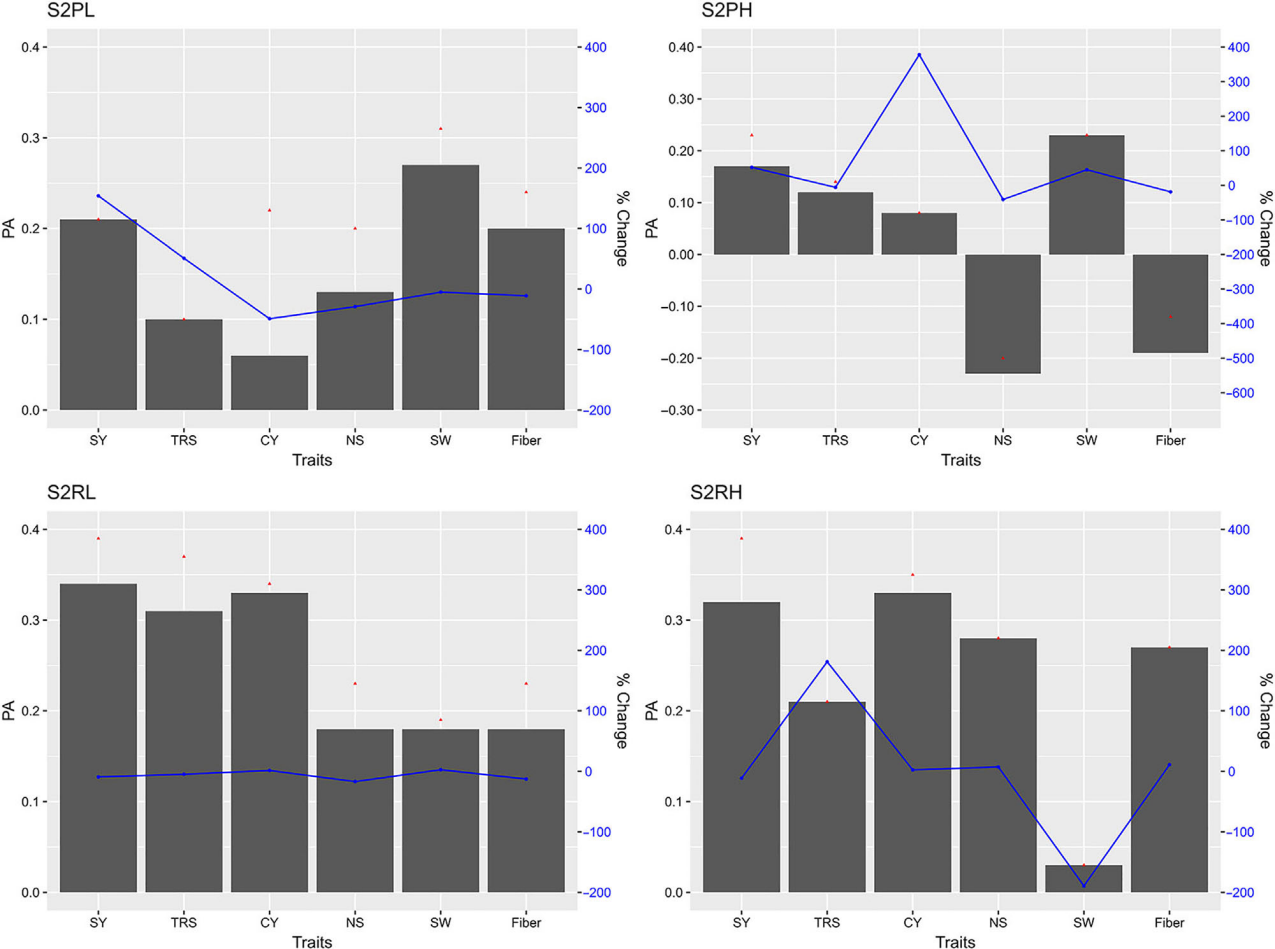
Predictive ability (PA) of GBLUP with significant single nucleotide variations (SNPs) as fixed effect (G + S) model and percentage change compared to genomic best linear unbiased prediction (GBLUP) model in stage 2 (S2) datasets. The bar graph, red point, and blue lines show the PA of G + S model, the highest PA, and percentage change of the PA by G + S model compared to GBLUP, respectively. CY, cane yield (Mg ha^−1^); Fiber, fiber content (%); H, heavy; L, light soil; NS, number of stalks (ha^−1^); P, plant cane; R, ratoon; SW, stem weight (Mg); SY, sugar yield (Mg ha^−1^); TRS, theoretical recoverable sugar (kg Mg^−1^).

**FIGURE 6:**
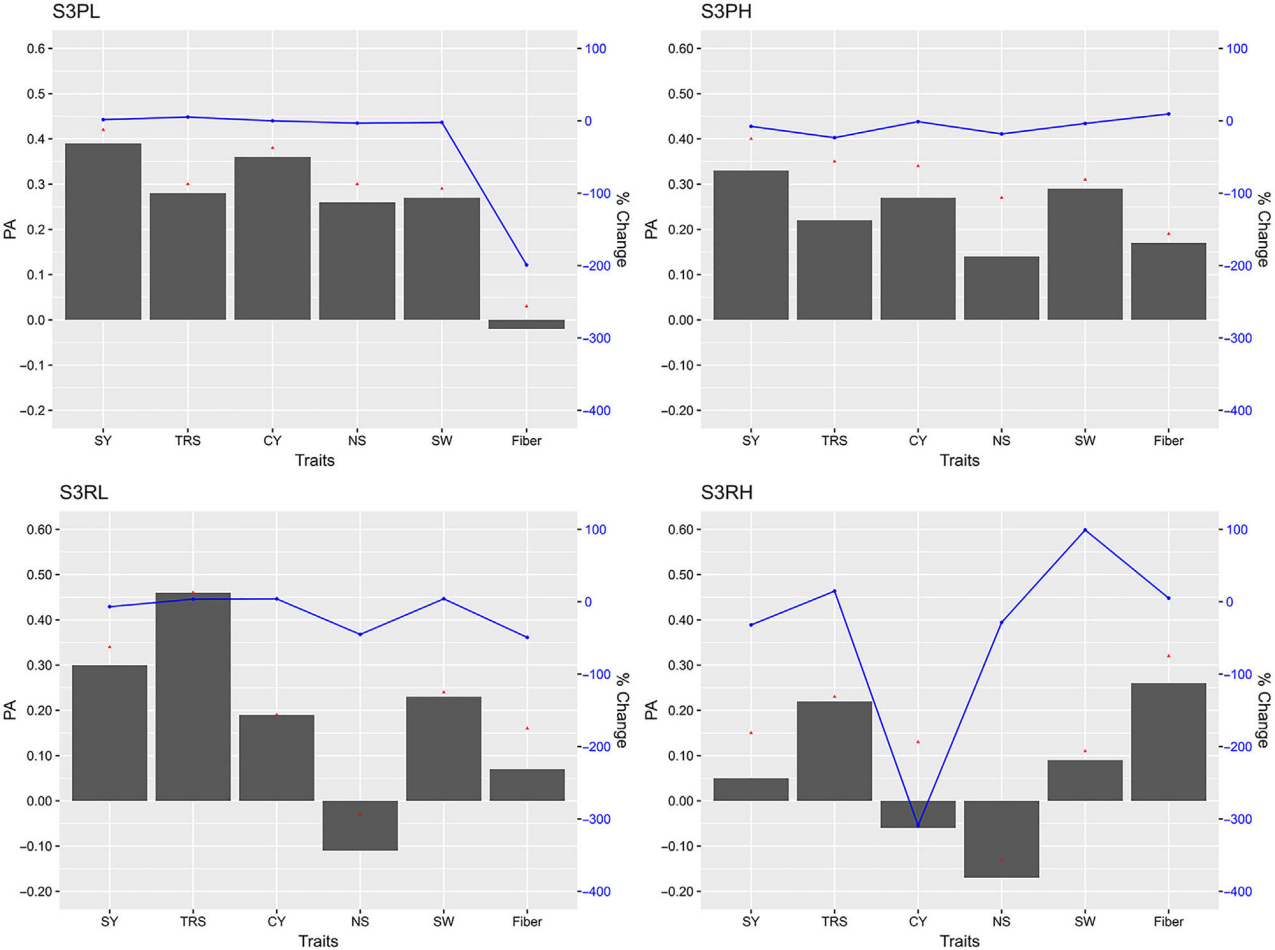
Predictive ability (PA) of with significant single nucleotide variations (SNPs) as fixed effect (G + S) model and percentage change compared to genomic best linear unbiased prediction (GBLUP) model in stage 4 (S3) datasets. The bar graph, red point, and blue lines show the PA of G + S model, the highest PA, and percentage change of the PA by G + S model compared to GBLUP, respectively. CY, cane yield (Mg ha^−1^); Fiber, fiber content (%); H, heavy; L, light soil; NS, number of stalks (ha^−1^); P, plant cane; R, ratoon; SW, stem weight (Mg); SY, sugar yield (Mg ha^−1^); TRS, theoretical recoverable sugar (kg Mg^−1^).

**FIGURE 7:**
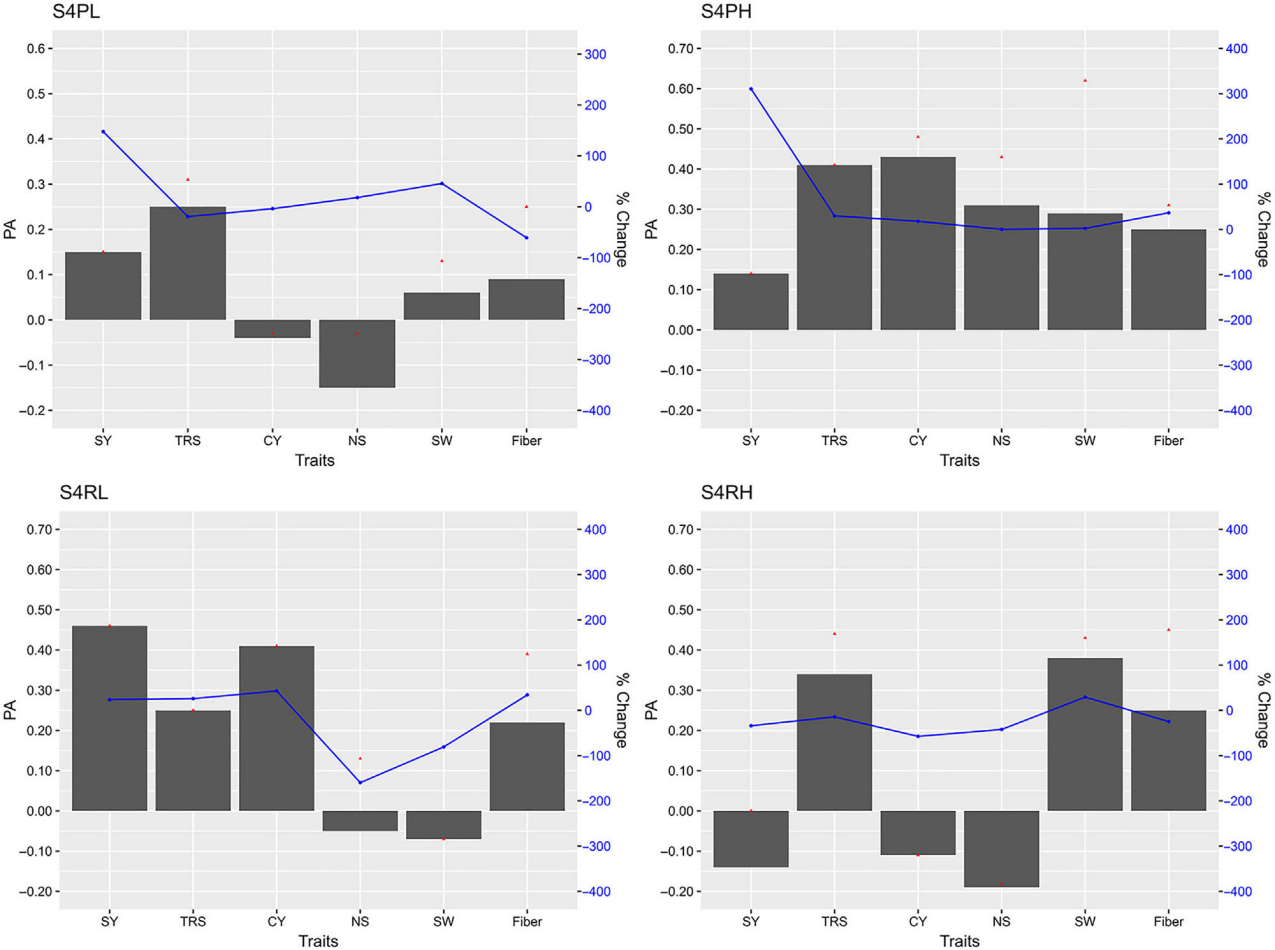
Predictive ability (PA) of with significant single nucleotide variations (SNPs) as fixed effect (G + S) model and percentage change compared to genomic best linear unbiased prediction (GBLUP) model in stage 4 (S4) datasets. The bar graph, red point, and blue lines show the PA of G + S model, the highest PA, and percentage change of the PA by G + S model compared to GBLUP, respectively. CY, cane yield (Mg ha^−1^); Fiber, fiber content (%); H, heavy; L, light soil; NS, number of stalks (ha^−1^); P, plant cane; R, ratoon; SW, stem weight (Mg); SY, sugar yield (Mg ha^−1^); TRS, theoretical recoverable sugar (kg Mg^−1^).

Significant SNPs improved the PA in some cases, whereas there was either no change or reduction in PA with the use of putative‐associated SNPs (Figures 5, 6, 7). With fivefold cross‐validation in S2PL, G + S had the highest PA for SY, which was a 154% increase over GBLUP (Figure 5, Table S26). In S2PH, the SNPs had a positive effect in predicting CY with higher accuracy by 378% increase over GBLUP, highest PA for TRS, SW, and Fiber in S2RH. At stage 3, G + S did not show much improvement over GBLUP but resulted in the highest PA for TRS and CY at S3RL (Figure 6, Table S27). At stage 4, fixed effect SNPs resulted in the highest PA for SY at S4PL, the highest PA for SY and CY with a significantly higher percentage increase over GBLUP in S4PH (Figure 7, Table S27). The G + S model accounted for the highest PA for SY, TRS, and SW at S4RL, with 23.62%–42. 84% increase in PA over GBLUP. In S4RH, significant SNPs did not improve the PA for the traits except for SW.

Inconsistent but more negative results were obtained with the fixed effect SNPs for the cross‐stage prediction of the traits (Table S28). Higher accuracy values were obtained with G + S model for TRS, CY, and SY with 18.74%, 104.98%, and 93.47% increase over GBLUP, respectively, at S5PL when predicted from S2L. Similarly, fixed effect SNPs improved the PAs of TRS (43.86%) and SW (90.77%) at S5PH with prediction from S2H. The PA for CY was increased exorbitantly higher (935.60%) with associated SNPs as a fixed effect in the model. Likewise, there was a 1494.28% and 1345.48% increase for SY and CY, respectively, with the G + S model when the performance of the clones at S5R2L was predicted from S2L. In cross‐stage predictions also, there was an increase in the PA for traits, except NS and Fiber at S5R2H from S2H.

#### 3.2.4 Multi‐trait genomic selection

**FIGURE 8:**
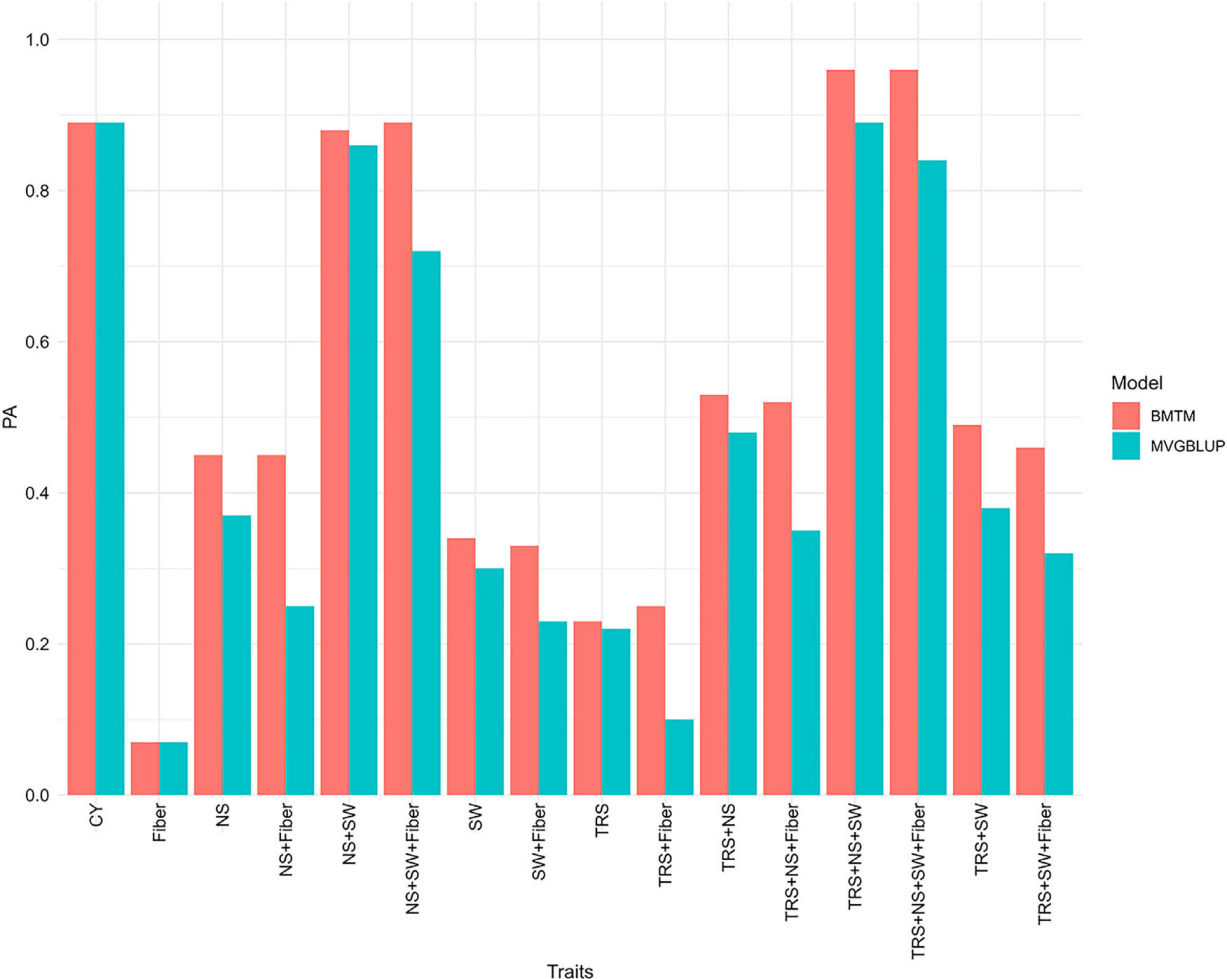
Multi‐trait genomic prediction of sugar yield accounting for various traits in Bayesian multi‐trait model and multi‐variate genomic best linear unbiased prediction (GBLUP) model in stage 2 in plant cane under light soil (S2PL). BMTM, Bayesian multi‐trait model; CY, cane yield (Mg ha^−1^); Fiber, fiber content (%); MVGBLUP, multi‐variate genomic best linear unbiased prediction; NS, number of stalks (ha^−1^); PA, predictive ability; SW, stem weight (Mg); TRS, theoretical recoverable sugar (kg Mg^−1^).

**FIGURE 9:**
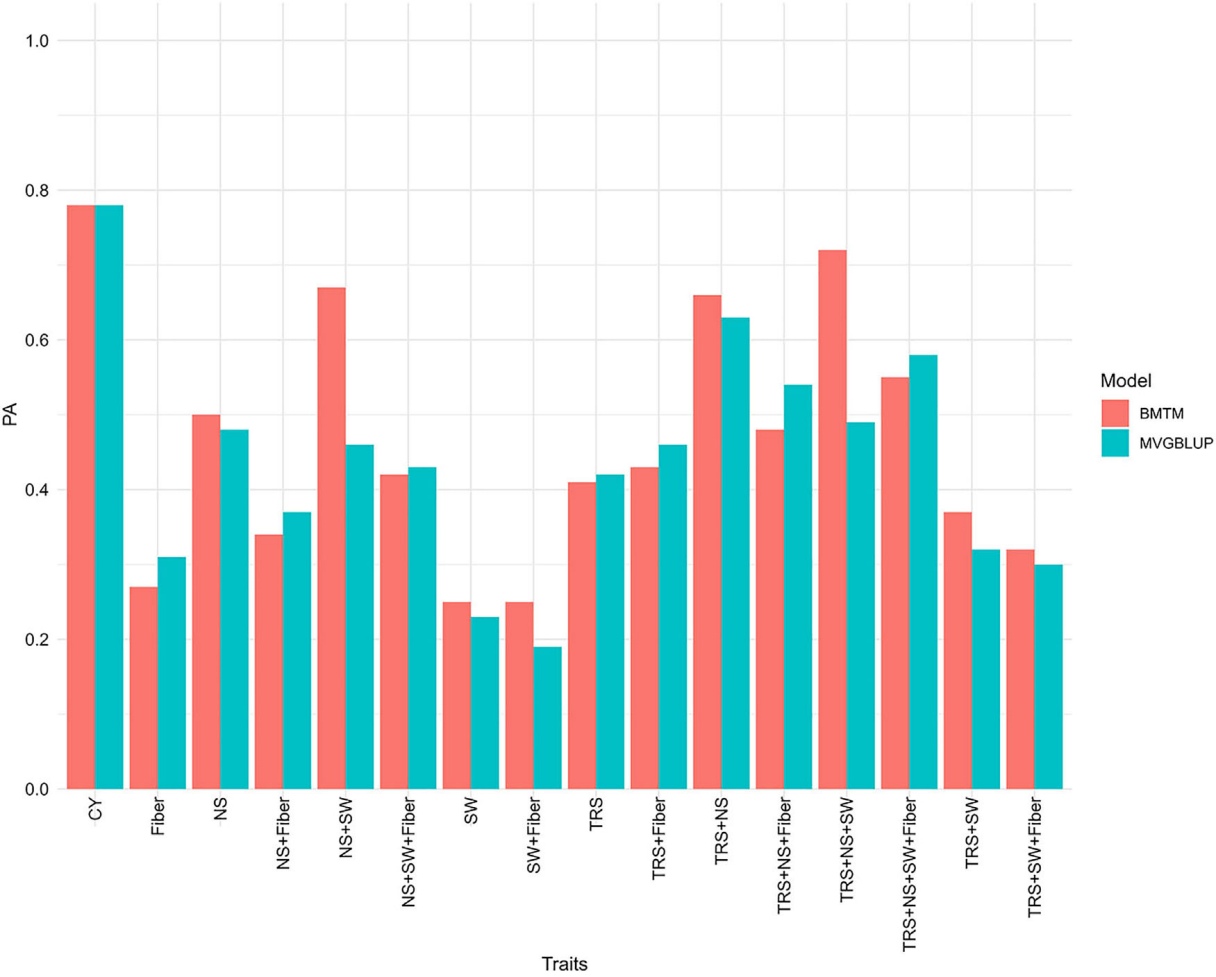
Genomic predictive ability (PA) of sugar yield accounting various traits in Bayesian multi‐trait model and multi‐variate genomic best linear unbiased prediction (GBLUP) model for prediction in stage 5 in plant cane under light soil (S5PL) from stage 2 in plant cane under light soil (S2PL). BMTM, Bayesian multi‐trait model; CY, cane yield (Mg ha^−1^); Fiber, fiber content (%); MVGBLUP, multi‐variate genomic best linear unbiased prediction; NS, number of stalks (ha^−1^); PA, predictive ability; SW, stem weight (Mg); TRS, theoretical recoverable sugar (kg Mg^−1^).

The PA in MTGS increased with the increase in the correlation of these traits with SY. Multi‐trait modeling with fivefold cross‐validation in S2PL showed a PA for SY (originally with a PA of 0.08) ranging from 0.07 with Fiber to 0.96 when TRS, NS, SW, and Fiber were all included in BMTM and from 0.00 to 0.84 with the same traits with multivariate GBLUP (MVGBLUP; Figure 8, Table S29). Using only TRS in the model, up to 0.23 (286.5% increase) PA was obtained. Similarly, when TRS and NS both were included in the prediction model, an accuracy of 0.48 (BMTM) to 0.53 (MVGBLUP) was achieved. On the other hand, the accuracy of multi‐trait modeling for cross‐stage prediction in S5PL using S2PL as the reference population for SY (originally with 0.14 PA) was the lowest (0.19 for MVGBLUP and 0.25 for BMTM) when SW and Fiber were considered, while the highest value of 0.78 was observed with CY as the predictor for both models (Figure 9, Table S29). Accounting TRS and NS, which had significantly positive correlation with SY at different stages (Table S30) in the multi‐trait models, resulted in a high PA of SY at 0.63 for MVGBLUP and 0.68 for BMTM.

#### 3.2.5 Effect of marker density on GP

**FIGURE 10:**
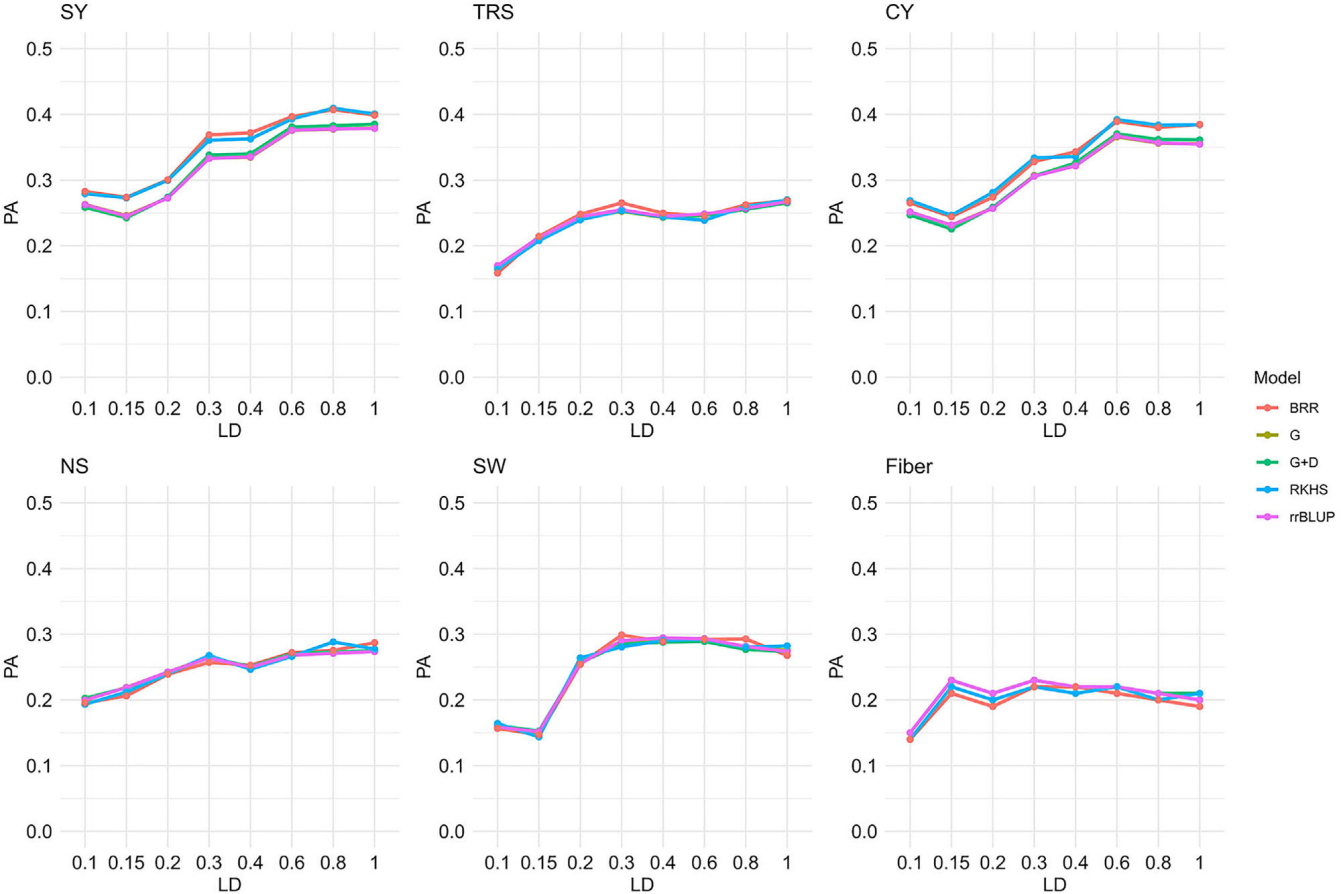
Predictive ability (PA) using five different models with different linkage disequilibrium (LD) pruning values in stage 3 in plant cane under light soil (S3PL). CY, cane yield (Mg ha^−1^); D, dominance; Fiber, fiber content (%); G, GBLUP; NS, number of stalks (ha^−1^); RKHS, reproducing kernel Hilbert space; rrBLUP; ridge regression BLUP; SW, stem weight (Mg); SY, sugar yield (Mg ha^−1^); TRS, theoretical recoverable sugar (kg Mg^−1^).

A comparative assessment of PA for six traits using different marker numbers, selected based on the LD between them, with five models, BRR, e(G)BLUP, RKHS, and rrBLUP suggested that the marker density significantly affected model performance. The PA generally increased with an increase in the number of markers; however, it plateaued for all the models at 18,012 (Figure 10). For NS and SW, the PA reached its maximum at 9091 markers and remained unchanged afterward. Prediction for Fiber showed the highest accuracy at 4399 (and 9091) markers. The number of markers for optimal PA also depended on the traits. For NS, SW, CY, and Fiber, all models predicted the highest accuracy with 15,592 SNPs after which it plateaued or started to decline. On the other hand, for maximum accuracy of prediction for TRS and SY, 18,012 SNPs were optimal. Across all models, however, the scenario was slightly different. For traits, such as TRS, NS, and Fiber, there was no significant difference in the PA beyond 6112 SNPs. Likewise, for SY, CY, and SW, the PAs were not significantly different after 9091 markers.

## 4 DISCUSSION

The availability of low‐cost, advanced genotyping technologies that generate abundant markers has facilitated the wide adoption of genomics‐assisted breeding in several plant breeding programs. The identification of multiple QTLs and SNPs associated with TRS, NS, SW, CY, SY, and Fiber in sugarcane indicates that these traits are genetically complex and governed by both large and small‐effect QTLs, thus making their utilization in MAS difficult. GS, as a genome‐wide molecular MAS, has shown to be promising in sugarcane (Islam et al., 2022, 2021; Satpathy et al., 2022; Yadav et al., 2021), which can be used as a powerful tool to select superior parents and/or progenies and enhance genetic gain in sugarcane.

Several factors, such as genetic architecture and heritability of the trait, training sample size, population structure, that is, relatedness of the training set with testing population, genes or loci associated with trait, genome size, ploidy level, gene action (additive/non‐additive), marker density, and statistical models affect prediction accuracy in GS (Bernardo, 2014; Islam et al., 2021; Maulana et al., 2021; Rutkoski et al., 2014; Yadav et al., 2021). In this study, traits with higher heritability, such as SW and TRS, exhibited higher prediction accuracies when compared to traits with lower heritability such as NS and CY. This suggests that a greater proportion of their phenotypic variation is explained by genetic factors, making them more suitable for predictions based on genomic information. Several previous studies have demonstrated a strong correlation between heritability and prediction accuracy (Kaler et al., 2022; Kwong et al., 2017). Highly heritable traits are generally controlled by a few major QTLs as compared to the multiple small‐effect genes controlling a trait with low heritability. Optimal GP for traits with low heritability such as NS and CY observed in our study can be achieved by increasing the TP size (*N*) to enhance the power of the models, which is influenced by *N* × *h* ^2^ (Bernardo, 2016).

Previous studies have suggested various GS models provide different prediction accuracy across crop species and traits (Bernardo, 2014; De Los Campos et al., 2010). In this study, we evaluated 20 parametric and two nonparametric models to examine their prediction reliability within and across various stages of sugarcane breeding programs in Louisiana. For most datasets, there was not much difference in the PA between GBLUP and Bayesian models. In agreement with our results, similar or very little difference was observed for PA between GBLUP and nonlinear Bayesian models (Deomano et al., 2020; Moser et al., 2009). On the other hand, extending additive GBLUP to incorporate dominance, epistasis, and average heterozygosity (eGBLUP) resulted in improvement of PA for some traits in a few datasets, for example, SY and Fiber in S3PL, but not in all datasets. In sugarcane, nonadditive genetic effects were considered significant for complex traits such as CY (Yadav et al., 2021). In addition to the potential reduction of inbreeding depression risk using models accounting for nonadditive effects and heterozygosity in commercial populations for traits such as CY (de Azeredo et al., 2016), dominance effects were considered important for selecting parents based on the GP of cross‐appraisal in clonal breeding programs (Werner et al., 2023). While Islam et al. (2022) observed significant differences in traits between the models with and without nonadditive effects in the model, Yadav et al. (2021) obtained higher PA for CY with eGBLUP but no improvement for SY and Fiber yield where nonadditive variance and heterozygosity were included. Only a slight increment in PA with eGBLUP was noted in other crops such as potato (Endelman et al., 2018), whereas de Bem Oliveira et al. (2019) observed no difference in PA with models considering additive and nonadditive genetic components in tetraploid blueberry for fruit firmness, fruit weight, and yield. The PA for these traits also did not improve with average heterozygosity incorporated into the GBLUP model, which was explained by the lack of variation in average heterozygosity among the genotypes.

The use of a relationship matrix including pedigree and genomic information (HBLUP) in GS models was shown to improve PA (Crossa et al., 2010; Sukumaran et al., 2017), especially when very precise pedigree data including several generations were available (Juliana et al., 2017). In our study, the HBLUP model outperformed other models for a few traits in both within‐stage cross‐fold validations and cross‐stage predictions in some datasets. Including pedigree can increase prediction performance where markers may not be sufficient to capture the genetic variations at the population and family levels (Velazco et al., 2019), otherwise the combination matrix may not be beneficial in mixed models due to information matrix redundancy (Albrecht et al., 2011). Also, pedigree‐based methods reportedly tend to overestimate the reliability of GS and PA (de Bem Oliveira et al., 2020; Gorjanc et al., 2015).

Nonparametric ML models are more flexible and adaptable to various data patterns with no requirement for predefined parameter specifications as the data determine the structure of the model. These models are useful especially when datasets are complex or where relationships are undefined (Montesinos‐López et al., 2022). In this study, ML methods such as RF and SVM performed better compared to other models, especially for the traits with low heritability such as NS. However, coincidence index, which signifies the potential of model to select better performing genotypes or discard the low‐performing genotypes, was comparable to or lower than other methods. Therefore, ML methods may not be the best choice for GS as they require higher computational time. This suggests if large‐scale genotyping is conducted on almost all clones included in field trials in the breeding program over several years (as was the case here), a GBLUP model will be sufficient for genetic evaluations of clones for traits with moderate to high heritability.

The accuracy has been reported to increase by incorporating major effect markers as fixed effect covariates in the prediction model in wheat (Sarinelli et al., 2019), rice (Anilkumar et al., 2023), and other crops (Chen et al., 2023; Kim et al., 2022). Similar results were obtained in sugarcane where Islam et al. (2021) found an increased prediction for brown rust disease resistance with the use of a major resistance gene *Bru*1 as a fixed effect. On the other hand, no significant difference in the accuracy was observed with the addition of the SNP markers as fixed‐effect covariates in corn and sorghum (Rice & Lipka, 2019). In our previous study also (Satpathy et al., 2022), mixed results were obtained for cane and sucrose yield traits with putatively associated SNPs as fixed effects in rrBLUP model. In the present study, the GBLUP model with GWAS‐derived SNPs as fixed effects (G + S) performed better with improved PA in a few datasets. For example, the G + S model significantly increased prediction of SY in S5PL, S5R1L, and S5R2L, TRS in S5PL and S5PH, CY in S5PH, and SW in S5R2H using S2L, S2PL, and S2H as the training set. However, such improvement was inconsistent across traits and datasets. A possible explanation is that the SNPs identified from GWAS study performed across different datasets were used as fixed effect covariates for all predictions. So, some of these SNP markers possibly are not strongly associated with a trait in a particular dataset. Therefore, the SNPs need to be validated before they can be effectively used as fixed effects in the model. While implementing GS in practical breeding, the choice of prediction models would depend on the heritability and genetic control of the trait(s) of interest as well as the availability of validated, small‐to‐large effect markers associated with the traits. However, if computational resources are not limited, we propose to use eGBLUP models with trait‐associated SNPs as fixed effects in the prediction models.

It is possible that the unbalanced nature of historical datasets where a few entries are represented over years (Table S31), especially at the early stage of selection where genotypic diversity is higher, can confound the accuracy of model training and thereby influence the model generalizability. Nevertheless, GRM estimated with high‐density marker data of nonoverlapping genotypes across years, defined target environments over time, and overlapping genotypes across selection stages (Table S33) can help model genetic covariances for genotypes repeated in different years, which allows effective utilization of multi‐year historical data (Bernal‐Vasquez et al., 2017). Successful utilization of historical breeding trial data in GP has been reported in different breeding programs including wheat (Dawson et al., 2013; Gonzalez et al., 2018; Sarinelli et al., 2019; Sneller et al., 2021). The PA values showed an increase for most traits with the advancement of stages. This was probably not due to the skewness of the traits toward higher values at later stages following selection, as the PA would depend primarily on GRM/marker effect rather than the absolute values. However, the lower range of variation in the dataset across environments (especially years) at the advanced stages may increase the PA for the traits.

GP can facilitate prediction of progeny performance across locations over years by improving the efficiency of multi‐environment testing in cultivar development pipeline as it helps to discard low‐ to mediocre‐performing breeding lines in the early stages, thus saving valuable time and resources (Atanda et al., 2021). Therefore, GP‐based sparse testing will be a viable approach to reduce the number of breeding lines yet keeping the same or even increasing the number of trial environments without increasing costs but maintaining the selection intensity in the early stages of evaluation (Montesinos‐López et al., 2023). In crops such as sugarcane where it takes ∼12 years from planting the seedlings in the field to potentially releasing a variety, genetic gain can be realized faster if the breeding cycle length can be reduced considering that optimal selection intensity has been realized by phenotypic or MAS of the traits of interest. Therefore, deployment of GS to select better performing clones as early as stage 1 or 2 will be very beneficial for the sugarcane industry. To this end, the performance of the clones at S5 was predicted as test population based only on their genotype using the models that accounted for the performance of the training set at S2 and S3.

In cross‐stage validation, PA was slightly higher when predicted from S3, which is replicated as against unreplicated S2 dataset. Expectedly, PA varied based on the traits and their heritability, population size, crop type, environment, and the models used. When S2 data combined across crop and soil were used, higher PAs of 0.44 for TRS, 0.42 for SW, and 0.51 for SY were obtained. Again, as was observed for within‐stage cross‐fold validations, the PAs were comparatively less for CY and NS at 0.30 and 0.17, respectively, in S5 trials predicted from S2. In a more practical sense, it will be beneficial to predict the performance of the clones at S5 at the plant cane crop in S2. In this scenario, PAs of 0.59 for SY, 0.61 for CY, 0.54 for NS, 0.32 for SW, and 0.50 for Fiber were obtained in S5PL. These results were comparable to those from other studies (Deomano et al., 2020; Gouy et al., 2013; Hayes et al., 2021; Yadav et al., 2021). Actual genetic gain obtained with the clones at S5 selected through phenotypic recurrent selection vis‐à‐vis that obtained using GEBV from S2 showed mostly improvement for the traits (Table S32). Considering the number of years taken for advancing these clones from S2 to S5 through recurrent selection (10 years) and through GEBV (7 years), the rate of gain was higher with GS for the SY attributing traits, with a 101% increase for TRS and 11% for SW compared to the conventional phenotypic selection. However, the rate was lower for NS, which was expected because of the low PA of the trait. In addition to shortening the breeding cycle, saving investments in land and planting resources via early selection of the clones is paramount.

The agronomic performance of a sugarcane clone in ratoon crops is an important consideration for its advancement/release as a variety in the US sugarcane industries, especially in Louisiana where it is only a 7‐ to 8‐month crop. Therefore, the performance of the clones in the ratoon crops was predicted from plant cane datasets early in the breeding cycle. When the combined dataset of S2 was used as the TP, the PAs for different traits in S5 ratoon crop irrespective of soil and crop were 0.14 for SY, 0.29 for TRS, 0.24 for NS and CY, 0.33 for SW, and 0.10 for Fiber. Similar prediction of sugarcane ratooning ability with an accuracy of 0.21 for NS and 0.31 for SY was observed for Florida clones with a fivefold cross‐validation approach (Islam et al., 2023). As expected, our results suggested that the performance prediction of the clones at S5 in light soil should be made from S2 light soil data and likewise for S5 heavy soil from S2 heavy soil data, where the PAs ranged from 0.05 to 0.31 for SY and from 0.44 to 0.66 for TRS instead of cross‐soil prediction. Prediction of the clones for all traits at S5 first ratoon crop was less accurate than in the plant cane and second ratoon crop under light soil when S2 combined data under light soil was used as TP.

Most GS studies in sugarcane reported so far used single‐trait GP models for predicting a trait of interest. However, PA of a trait has been shown to improve by incorporating multiple traits in the prediction models in crops such as wheat (Gill et al., 2023; Hayes et al., 2017; Lado et al., 2018; Michel et al., 2018; Shahi et al., 2022; Zhang‐Biehn et al., 2021) and potato (Ortiz et al., 2023). For example, multi‐trait GP improved the accuracy by nearly 100% (0.75) for baking absorption and by 63% for loaf volume in wheat compared to single‐trait prediction (Gill et al., 2023). We observed that MTGS improved PA for SY, although the extent of improvement varied depending on the extent of correlations between SY and the traits incorporated in the model (Table S29). For example, there was very little improvement in PA when Fiber was used in the model in contrast to significantly higher improvement when highly correlated traits such as CY and TRS were used. In sugarcane, traits such as NS and TRS can be available early in the growing season and using them as early as S2 in GS to predict SY for S5 can be very useful. This was evident from the increase in prediction of SY with single‐trait model (0.30) to 0.50 and 0.41 using a multi‐trait model with NS and TRS, respectively, and up to 0.66 when both traits were considered, for cross‐stage prediction from stage 2 as training set to stage 5 as test population.

Marker density has also been shown to influence PA (Norman et al., 2018; Zhang et al., 2019). While the big sugarcane genome may warrant a large SNP dataset generated through next‐generation sequencing (NGS)‐based genotyping tools, it is important to determine the marker density that is required to obtain optimally high PA considering the high LD of sugarcane and the fact that most markers are phenotypically irrelevant or neutral (Weber et al., 2023). Our evaluation showed a generally linear relationship where PA increased with an increase in the number of markers. It is possible that more markers distributed across chromosomes could accurately capture most contributing QTLs, ultimately leading to an increased prediction. The marker density at which the PA plateaued depended on the traits studied. We found that 9091 markers for highly heritable traits such as TRS, SW, and SY, and 15,592 for low‐heritable traits such as NS and CY were sufficient for optimal GP accuracy. This result is comparable with a study in blueberry where 10,000 SNPs were found optimum for GP (de Bem Oliveira et al., 2020). While such a high marker number is expected for genome coverage in a polyploid crop such as sugarcane and blueberry, other studies in sugarcane suggested that 5000 (Islam et al., 2022) and 3000 (Islam et al., 2021) SNPs were enough for optimal PA. However, both these reports were based on brown rust and orange rust resistance with reportedly high heritability. These results suggested that the number of markers used in GS of crops with large LD such as sugarcane can be reduced without compromising its performance as long as the markers are evenly represented in the LD blocks across the genome (Ballesta et al., 2020; Silva et al., 2018). Selection of optimal subsets of markers for specific traits has also been proposed to increase GP accuracy (Alemu et al., 2023; Filho et al., 2019). In addition, inclusion of run of heterozygosity and continuous genotype of the clones in the models has shown potential for the improvement of PAs for the trait(s) of the interest in sugarcane (Yadav et al., 2024).

## 5 CONCLUSIONS

GS showed great promise to increase the rate of genetic gain per year at a lower cost and in less time compared to the conventional recurrent selection method for key traits in sugarcane variety development programs by considering GEBVs for these traits early in the breeding cycle. Taken together, our resultsn sugarcane and previous findings in sugarcane and other polyploid crops reflected on the successful implementation of GS in sugarcane breeding program where using historical data in the TP set is beneficial to predict GEBV of the clones early to facilitate their testing in multi‐environment yield trials in subsequent years. Specifically, selection of better performing clones or elimination of low performers at S2, especially at S2P, can save at least 2–3 years of S4 testing, thus potentially enhancing the rate of genetic gain by reducing the breeding cycle length. Alternatively, the selected clones at S2P can be grown in S3 nurseries to increase the population for testing directly at S5 to select the potential variety. However, in a complex crop such as sugarcane, it is better to have data on a larger TP for better fit of the models to be used for GP, especially for traits with low heritability and complex genetic architecture. Multi‐trait GP using NS and SW, available early at S2, is a better strategy for improving PA of SY.

## AUTHOR CONTRIBUTIONS

**Dipendra Shahi**: Formal analysis; investigation; methodology; software; writing—original draft. **James Todd**: Data curation; resources; writing—review and editing. **Kenneth Gravois**: Data curation; resources; writing—review and editing. **Anna Hale**: Data curation; resources; writing—review and editing. **Brayden Blanchard**: Data curation; resources; writing—review and editing. **Collins Kimbeng**: Data curation; resources; writing—review and editing. **Michael Pontif**: Data curation; resources; writing—review and editing. **Niranjan Baisakh**: Conceptualization; data curation; formal analysis; funding acquisition; investigation; project administration; resources; writing—review and editing.

## CONFLICT OF INTEREST STATEMENT

The authors declare no conflicts of interest.

## Supporting information




**Table S1**. Summary statistics for six cane and sugar yield traits at different selection stages (2, 3, 4, and 5) SY, sugar yield (Mg ha^−1^); TRS, theoretical recoverable sugar (kg Mg^−1^); CY, cane yield (Mg ha^−1^); NS, number of stalks ha^−1^; SW, stalk weight (kg); P, plant cane; R, ratoon; R1, first ratoon; R2, second ratoon; H, heavy soil; L, light soil.
**Table S2**. Correlation among six cane and sugar yield traits at different selection stages (2, 3, 4, and 5) SY, sugar yield (Mg ha^−1^); TRS, theoretical recoverable sugar (kg Mg^−1^); CY (Mg ha^−1^), cane yield (Mg ha^−1^); NS, number of stalks ha^−1^; SW, stalk weight (kg); Fiber, fiber (%); S, stage; P, plant cane; R, ratoon; H, heavy soil; L, light soil.
**Table S3**. Narrow‐sense and broad‐sense heritability (H^2^) of six cane and sugar yield traits
SY, sugar yield (Mg ha^−1^); TRS, theoretical recoverable sugar (kg Mg^−1^); CY (Mg ha^−1^), cane yield (Mg ha^−1^); NS, number of stalks ha^−1^; SW, stalk weight (kg); Fiber, fiber (%); S, stage; P, plant cane; R, ratoon; H, heavy soil; L, light soil. h^2^_p, narrow‐sense heritability (pedigree); h^2^_m, narrow‐sense heritability (marker); H^2^, broad‐sense heritability; SE, standard error.
**Table S4**. Predictive ability of six cane and sugar yield traits at stage 2 in plant cane crop under light soil (S2PL) SY, sugar yield (Mg ha^−1^); TRS, theoretical recoverable sugar (kg Mg^−1^); CY (Mg ha^−1^), cane yield (Mg ha^−1^); NS, number of stalks ha^−1^; SW, stalk weight (kg); Fiber, fiber (%); PA, predictive ability; TCI, top coincidence index; BCI, bottom coincidence index.
**Table S5**. Predictive ability of six cane and sugar yield traits in stage 2 plant cane crop under heavy soil (S2PH) SY, sugar yield (Mg ha^−1^); TRS, theoretical recoverable sugar (kg Mg^−1^); CY (Mg ha^−1^), cane yield (Mg ha^−1^); NS, number of stalks ha^−1^; SW, stalk weight (kg); Fiber, fiber (%); PA, predictive ability; TCI, top coincidence index; BCI, bottom coincidence index.
**Table S6**. Predictive ability of six cane and sugar yield traits in stage 2 ratoon crop under light soil (S2RL) SY, sugar yield (Mg ha^−1^); TRS, theoretical recoverable sugar (kg Mg^−1^); CY (Mg ha^−1^), cane yield (Mg ha^−1^); NS, number of stalks ha^−1^; SW, stalk weight (kg); Fiber, fiber (%); PA, predictive ability; TCI, top coincidence index; BCI, bottom coincidence index.
**Table S7**. Predictive ability of six cane and sugar yield traits in stage 2 ratoon under heavy soil (S2RH) SY, sugar yield (Mg ha^−1^); TRS, theoretical recoverable sugar (kg Mg^−1^); CY (Mg ha^−1^), cane yield (Mg ha^−1^); NS, number of stalks ha^−1^; SW, stalk weight (kg); Fiber, fiber (%); PA, predictive ability; TCI, top coincidence index; BCI, bottom coincidence index.
**Table S8**. Predictive ability of six cane and sugar yield traits in stage 3 plant cane under light soil (S3PL) SY, sugar yield (Mg ha^−1^); TRS, theoretical recoverable sugar (kg Mg^−1^); CY (Mg ha^−1^), cane yield (Mg ha^−1^); NS, number of stalks ha^−1^; SW, stalk weight (kg); Fiber, fiber (%); PA, predictive ability; TCI, top coincidence index; BCI, bottom coincidence index.
**Table S9**. Predictive ability of six cane and sugar yield traits in stage 3 plant cane under heavy soil (S3PH) SY, sugar yield (Mg ha^−1^); TRS, theoretical recoverable sugar (kg Mg^−1^); CY (Mg ha^−1^), cane yield (Mg ha^−1^); NS, number of stalks ha^−1^; SW, stalk weight (kg); Fiber, fiber (%); PA, predictive ability; TCI, top coincidence index; BCI, bottom coincidence index.
**Table S10**. Predictive ability of six cane and sugar yield traits in stage 3 ratoon under light soil (S3RL) SY, sugar yield (Mg ha^−1^); TRS, theoretical recoverable sugar (kg Mg^−1^); CY (Mg ha^−1^), cane yield (Mg ha^−1^); NS, number of stalks ha^−1^; SW, stalk weight (kg); Fiber, fiber (%); PA, predictive ability; TCI, top coincidence index; BCI, bottom coincidence index.
**Table S11**. Predictive ability of six cane and sugar yield traits in stage 3 ratoon under heavy soil (S3RH) SY, sugar yield (Mg ha^−1^); TRS, theoretical recoverable sugar (kg Mg^−1^); CY (Mg ha^−1^), cane yield (Mg ha^−1^); NS, number of stalks ha^−1^; SW, stalk weight (kg); Fiber, fiber (%); PA, predictive ability; TCI, top coincidence index; BCI, bottom coincidence index.
**Table S12**. Predictive ability of six cane and sugar yield traits in stage 4 plant cane under light soil (S4PL) SY, sugar yield (Mg ha^−1^); TRS, theoretical recoverable sugar (kg Mg^−1^); CY (Mg ha^−1^), cane yield (Mg ha^−1^); NS, number of stalks ha^−1^; SW, stalk weight (kg); Fiber, fiber (%); PA, predictive ability; TCI, top coincidence index; BCI, bottom coincidence index.
**Table S13**. Predictive ability of six cane and sugar yield traits in stage 4 plant cane under heavy soil (S4PH) SY, sugar yield (Mg ha^−1^); TRS, theoretical recoverable sugar (kg Mg^−1^); CY (Mg ha^−1^), cane yield (Mg ha^−1^); NS, number of stalks ha^−1^; SW, stalk weight (kg); Fiber, fiber (%); PA, predictive ability; TCI, top coincidence index; BCI, bottom coincidence index.
**Table S14**. Predictive ability of six cane and sugar yield traits in stage 4 ratoon under light soil (S4RL) SY, sugar yield (Mg ha^−1^); TRS, theoretical recoverable sugar (kg Mg^−1^); CY (Mg ha^−1^), cane yield (Mg ha^−1^); NS, number of stalks ha^−1^; SW, stalk weight (kg); Fiber, fiber (%); PA, predictive ability; TCI, top coincidence index; BCI, bottom coincidence index.
**Table S15**. Predictive ability of six cane and sugar yield traits in stage 4 ratoon under heavy soil (S4RH) SY, sugar yield (Mg ha^−1^); TRS, theoretical recoverable sugar (kg Mg^−1^); CY (Mg ha^−1^), cane yield (Mg ha^−1^); NS, number of stalks ha^−1^; SW, stalk weight (kg); Fiber, fiber (%); PA, predictive ability; TCI, top coincidence index; BCI, bottom coincidence index.
**Table S16**. Predictive ability of six cane and sugar yield traits in stage 5 using stage 2 (S2) as training dataset SY, sugar yield (Mg ha^−1^); TRS, theoretical recoverable sugar (kg Mg^−1^); CY (Mg ha^−1^), cane yield (Mg ha^−1^); NS, number of stalks ha^−1^; SW, stalk weight (kg); Fiber, fiber (%); PA, predictive ability; TCI, top coincidence index; BCI, bottom coincidence index.
**Table S17**. Predictive ability of six cane and sugar yield traits in stage 5 using stage 2 plant cane (S2P) as training dataset SY, sugar yield (Mg ha^−1^); TRS, theoretical recoverable sugar (kg Mg^−1^); CY (Mg ha^−1^), cane yield (Mg ha^−1^); NS, number of stalks ha^−1^; SW, stalk weight (kg); Fiber, fiber (%); PA, predictive ability; TCI, top coincidence index; BCI, bottom coincidence index.
**Table S18**. Predictive ability of six cane and sugar yield traits in stage 5 using stage 2 under light soil (S2L) as training dataset SY, sugar yield (Mg ha^−1^); TRS, theoretical recoverable sugar (kg Mg^−1^); CY (Mg ha^−1^), cane yield (Mg ha^−1^); NS, number of stalks ha^−1^; SW, stalk weight (kg); Fiber, fiber (%); PA, predictive ability; TCI, top coincidence index; BCI, bottom coincidence index.
**Table S19**. Predictive ability of six cane and sugar yield traits in stage 5 using stage 2 plant cane under light soil (S2PL) SY, sugar yield (Mg ha^−1^); TRS, theoretical recoverable sugar (kg Mg^−1^); CY (Mg ha^−1^), cane yield (Mg ha^−1^); NS, number of stalks ha^−1^; SW, stalk weight (kg); Fiber, fiber (%); PA, predictive ability; TCI, top coincidence index; BCI, bottom coincidence index.
**Table S20**. Predictive ability of six cane and sugar yield traits in stage 5 using second line under heavy soil (S2H) as training dataset SY, sugar yield (Mg ha^−1^); TRS, theoretical recoverable sugar (kg Mg^−1^); CY (Mg ha^−1^), cane yield (Mg ha^−1^); NS, number of stalks ha^−1^; SW, stalk weight (kg); Fiber, fiber (%); PA, predictive ability; TCI, top coincidence index; BCI, bottom coincidence index.
**Table S21**. Predictive ability of six cane and sugar yield traits in stage 5 using stage 2 plant cane under heavy soil (S2PH) as training dataset SY, sugar yield (Mg ha^−1^); TRS, theoretical recoverable sugar (kg Mg^−1^); CY (Mg ha^−1^), cane yield (Mg ha^−1^); NS, number of stalks ha^−1^; SW, stalk weight (kg); Fiber, fiber (%); PA, predictive ability; TCI, top coincidence index; BCI, bottom coincidence index.
**Table S22**. Predictive ability of cane and sugar yield six traits in stage 5 using stage 2 ratoon under light soil (S2RL) as training dataset SY, sugar yield (Mg ha^−1^); TRS, theoretical recoverable sugar (kg Mg^−1^); CY (Mg ha^−1^), cane yield (Mg ha^−1^); NS, number of stalks ha^−1^; SW, stalk weight (kg); Fiber, fiber (%); PA, predictive ability; TCI, top coincidence index; BCI, bottom coincidence index.
**Table S23**. Predictive ability of six cane and sugar yield traits in stage 5 using stage 2 combined ratoon under heavy soil (S2RH) as training dataset SY, sugar yield (Mg ha^−1^); TRS, theoretical recoverable sugar (kg Mg^−1^); CY (Mg ha^−1^), cane yield (Mg ha^−1^); NS, number of stalks ha^−1^; SW, stalk weight (kg); Fiber, fiber (%); PA, predictive ability; TCI, top coincidence index; BCI, bottom coincidence index.
**Table S24**. Predictive ability of six cane and sugar yield traits in stage 5 using stage 2 plant cane (S2P) as training dataset SY, sugar yield (Mg ha^−1^); TRS, theoretical recoverable sugar (kg Mg^−1^); CY (Mg ha^−1^), cane yield (Mg ha^−1^); NS, number of stalks ha^−1^; SW, stalk weight (kg); Fiber, fiber (%); PA, predictive ability; TCI, top coincidence index; BCI, bottom coincidence index.
**Table S25**. Predictive ability of six cane and sugar yield traits in stage 5 using stage 2 ratoon (S2R) as training dataset SY, sugar yield (Mg ha^−1^); TRS, theoretical recoverable sugar (kg Mg^−1^); CY (Mg ha^−1^), cane yield (Mg ha^−1^); NS, number of stalks ha^−1^; SW, stalk weight (kg); Fiber, fiber (%); PA, predictive ability; TCI, top coincidence index; BCI, bottom coincidence index.
**Table S26**. Significant SNPs used for G+S genomic selection model for six cane and sugar yield traits SY, sugar yield (Mg ha^−1^); TRS, theoretical recoverable sugar (kg Mg^−1^); CY, cane yield Mg ha^−1^); NS, number of stalks ha^−1^; SW, stem weight (kg); Fiber (%).
**Table S27**. Predictive ability of six cane and sugar yield traits with GWAS‐identified SNPs as fixed effect in GBLUP (G+S) model within a stage
SY, sugar yield (Mg ha^−1^); TRS, theoretical recoverable sugar (kg Mg^−1^); CY, cane yield (Mg ha^−1^); NS, number of stalks ha^−1^; SW, stalk weight (kg); Fiber (%); S, stage; P, plant cane; R, ratoon; L, light; H, heavy.
**Table S28**. Predictive ability of six cane and sugar yield traits with GWAS‐identified SNPs as fixed effect in GBLUP (G+S) model across stages S, stage; P, plant cane; R, ratoon; L, light; H, heavy.
**Table S29**. Multi‐trait genomic prediction of sugar yield accounting various traits in Bayesian multi‐trait model and multi‐variate GBLUP model SY, sugar yield (Mg ha^−1^); TRS, theoretical recoverable sugar (kg Mg^−1^); CY, cane yield (Mg ha^−1^); NS, number of stalks ha^−1^; SW, stalk weight (kg); Fiber (%); S, stage; P, plant cane; R, ratoon; L, light; H, heavy; BMTM, Bayesian multi‐trait model; MVGBLUP, multi‐variate genomic best linear unbiased prediction.
**Table S30**. Correlations among six cane and sugar yield traits between stages 2 and 5 * Significant at P≤0.05; ** significant at P≤0.01; *** significant at P≤0.001 SY, sugar yield (Mg ha^−1^); TRS, theoretical recoverable sugar (kg Mg^−1^); CY, cane yield (Mg ha^−1^); NS, number of stalks ha^−1^; SW, stalk weight (Mg); Fiber (%).
**Table S31**. Sugarcane genotypes tested at different environments (locations/year) in different selection stages.
**Table S32**. Rate of genetic gain of sugar yield attributing traits using recurrent phenotypic selection of clones vis‐à‐vis genomic selection in Louisiana sugarcane TRS, theoretical recoverable sugar (kg Mg^−1^); NS, number of stalks ha^−1^, SW, stalk weight (kg); S2, stage 2; S5, stage 5.
**Table S33**. Sugarcane genotypes common between different selection stages (numbers depicted in Venn diagram).

## Data Availability

All data supporting the findings of this study are available in the paper and its Supporting Information. The raw phenotype and genotype data used in this study can be accessed at https://agdatacommons.nal.usda.gov/articles/dataset/Louisiana_Sugarcane_yield_data_2010‐2021_collected_for_the_Louisiana_Variety_Development_Program/27248787/1 and https://agdatacommons.nal.usda.gov/articles/dataset/Data_from_Exploiting_Historical_Agronomic_Data_to_Develop_Genomic_Prediction_Strategies_for_Early_Clonal_Selection_in_the_Louisiana_Sugarcane_Variety_Development_Program/27679047?file=50390142, respectively.
